# Effects of Synbiotics on the Fecal Microbiome and Metabolomic Profiles of Healthy Research Dogs Administered Antibiotics: A Randomized, Controlled Trial

**DOI:** 10.3389/fvets.2021.665713

**Published:** 2021-05-26

**Authors:** Jacqueline C. Whittemore, Joshua M. Price, Tamberlyn Moyers, Jan S. Suchodolski

**Affiliations:** ^1^Small Animal Clinical Sciences, College of Veterinary Medicine, University of Tennessee, Knoxville, TN, United States; ^2^Office of Information Technology, University of Tennessee, Knoxville, TN, United States; ^3^The Gastrointestinal Laboratory, Small Animal Clinical Sciences, Texas A&M University, College Station, TX, United States

**Keywords:** antibiotic-associated diarrhea, dysbiosis, deoxycholic acid, D-erythro-sphingosine, bile acid diarrhea, probiotic, indole, propanediol

## Abstract

**Background:** Antibiotic-associated gastrointestinal signs occurred in 100% of dogs administered enrofloxacin with metronidazole in a previous study, and signs partially were mitigated by synbiotics. The objective of this randomized, double-blinded, placebo-controlled trial was to compare the fecal microbiome and metabolome of dogs administered enrofloxacin and metronidazole, followed by either a placebo or a bacterial/yeast synbiotic combination.

**Methods:** Twenty-two healthy research dogs were randomized to two treatment groups. There were three study periods: baseline, treatment, and washout. Dogs were administered enrofloxacin (10 mg/kg qd) and metronidazole (12.5 mg/kg BID), followed 1 h later by placebo or a commercially-available synbiotic combination (BID), per os for 21 days with reevaluation 56 days thereafter. Fecal samples were collected on days 5–7 (baseline), 26–28, and 82–84. The fecal microbiome was analyzed by qPCR and sequencing of 16S rRNA genes; time-of-flight mass spectrometry was used to determine metabolomic profiles. Split plot repeated measures mixed model ANOVA was used to compare results between treatment groups. *P* < 0.05 was considered significant, with Benjamini and Hochberg's False Discovery Rate used to adjust for multiple comparisons.

**Results:** Alpha diversity metrics differed significantly over time in both treatment groups, with incomplete recovery by days 82–84. Beta diversity and the dysbiosis index differed significantly over time and between treatment groups, with incomplete recovery at days 82–84 for dogs in the placebo group. Significant group-by-time interactions were noted for 15 genera, including *Adlercreutzia, Bifidobacterium, Slackia, Turicibacter, Clostridium* (including *C. hiranonis*) [*Ruminococcus*], *Erysipelotrichaceae_g_*, [*Eubacterium*], and *Succinivibrionaceae_g_*. Concurrent group and time effects were present for six genera, including *Collinsella, Ruminococcaceae_g*_, and *Prevotella*. Metabolite profiles differed significantly by group-by-time, group, and time for 28, 20, and 192 metabolites, respectively. These included short-chain fatty acid, bile acid, tryptophan, sphingolipid, benzoic acid, and cinnaminic acid metabolites, as well as fucose and ethanolamine. Changes in many taxa and metabolites persisted through days 82–84.

**Conclusion:** Antibiotic administration causes sustained dysbiosis and dysmetabolism in dogs. Significant group-by-time interactions were noted for a number of taxa and metabolites, potentially contributing to decreased antibiotic-induced gastrointestinal effects in dogs administered synbiotics.

## Introduction

Adverse antibiotic-induced gastrointestinal signs (AAGS) have been described in a variety of species, including people, cats, and dogs (1–5). Clinical signs are believed to stem primarily from derangement of the gastrointestinal microbiome, with broad-spectrum antibiotic regimens associated with an increased risk of AAGS (1). Administration of synbiotics, commercial combinations of probiotics and prebiotics, 1 h after antibiotic administration significantly decreases the occurrence and severity of AAGS in healthy cats and dogs (2, 5).

Marked reductions in fecal alpha and beta diversity have been identified during antibiotic administration in cats (4, 6) and dogs (7), with derangements in individual taxa and metabolite profiles identified up to 603 days after antibiotic discontinuation in cats. Furthermore, derangements significantly differed between cats administered antibiotics with a placebo vs. a synbiotic, as did the development of AAGS, suggesting a potential mechanism for AAGS reduction. The purpose of this randomized, double-blinded, placebo-controlled trial was to characterize and compare changes in the fecal microbiome and metabolomic profiles of healthy research dogs administered enrofloxacin and metronidazole, followed 1 h later by either a placebo or a bacterial/yeast synbiotic combination.

## Materials and Methods

### Study Design

**Figure 1:**
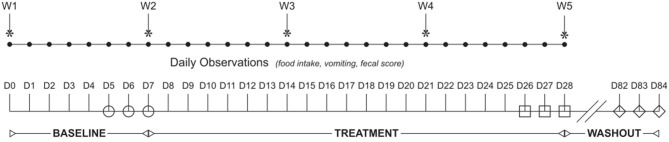
Study design flowchart. The study spanned 84 days (D1–84) and was broken into three study periods: baseline (D1–D7), treatment (D8–D28), and washout (D29–84). Dogs were randomized to receive enrofloxacin (10 mg/kg qd) and metronidazole (12.5 mg/kg BID), followed 1 h later by placebo or a synbiotic combination (BID) PO during treatment. Feces were collected from each dog once daily on the last 3 days of baseline (open circles), treatment (open squares), and recovery (open diamonds).

Fecal samples collected during the first phase of a previously reported randomized, double-blinded, placebo-controlled, 2-way, 2-period, cross-over study with a 8-week washout period (5) were used for this study (Figure 1). The study protocol was approved by the Institutional Animal Care and Use Committee of the University of Tennessee, Knoxville (protocol number 2544) and performed in compliance with “The Guide for the Care and Use of Laboratory Animals” in laboratory animal facilities that are AAALAC certified and exceed NIH standards of care.

Briefly, 22 healthy research dogs, randomized using a random number sequence to two treatment groups after stratification by breed, were included in the study. Each group contained 11 dogs: six female intact hound dogs and five male castrated beagles. All dogs were 1 year old. Median weight was 9.3 kg (range, 7.3–21.0 kg) for dogs in the placebo group and 9.3 kg (range, 7.8–18.5 kg) for dogs in the synbiotic group.

After a 1-week baseline, each dog was administered study medications for 21 days. Each dog received enrofloxacin (Baytril Taste Tabs®, Catalog# 08711391 and 08711405, Bayer Corporation, Shawnee Mission, KS, USA) 10 mg/kg q24h and metronidazole (Teva Pharmaceuticals USA, Inc., NDC# 50111-0333, North Wales, PA, USA) 12.5 mg/kg q12h per os in 13 gm of canned commercial dog food, after which each dog was given its ration of commercial dry dog food. One hour after antibiotic administration, each dog was administered two chewable tablets per os, containing either placebo or the probiotic/synbiotic combination as per group assignment. The probiotic/synbiotic combination consisted of one chewable multi-strain bacterial probiotic tablet (Proviable®-*Forte*, Catalog# PROVFTCHW180, Nutramax Laboratories Veterinary Sciences, Inc., Lancaster, SC, USA) plus one chewable yeast synbiotic tablet (Mycequin®, Catalog# MYCEQUIN144, Nutramax Laboratories Veterinary Sciences, Inc., Lancaster, SC, USA). Each tablet of the probiotic was formulated to contain 1 × 10^10^ cfu of a proprietary mixture of *Bifidobacterium bifidum, Enterococcus faecium, Streptococcus thermophilus*, and *Lactobacillus acidophilus, bulgaricus, casei*, and *plantarum*. Each tablet of the synbiotic was formulated to contain 1 × 10^10^ cfu of a proprietary strain of *Saccharomyces boulardii* and the prebiotic beta-glucan. Placebo tablets, provided by the manufacturer, did not differ in shape, size, smell, or flavoring from the probiotic and synbiotic tablets.

### Fecal Samples

First morning naturally-voided fecal samples were collected daily for 3 days from each dog at each time point (days 5–7, 26–28, and 82–84) to minimize the effects of daily variation and differential distribution of bacterial groups and metabolites within individual fecal samples on results. A 2-g sample was taken from the center portion of each fecal sample and subdivided into two aliquots, placed into individual 2 mL cryovials, and immediately frozen at −80°C pending completion of data collection. Samples for each dog from each time point (days 5–7, 26–28, and 82–84) were combined directly prior to sample analysis to generate pooled samples for microbiome and metabolomic analysis.

One dog in the placebo group was removed from treatment after 1 week of antibiotics due to excessive weight loss with associated hyporexia and vomiting. As a result, fecal samples from only 10 dogs in the placebo group were available for days 26–28.

### Microbiome Analysis

Fecal microbiome analysis was performed as per a previous study (7). To summarize, genomic DNA was extracted from 100 mg of feces from each pooled sample using a commercially available kit according to manufacturer's protocol (PowerSoil®, Catalog #12888-100, Mo Bio Laboratories, Carlsbad, CA, USA). Amplification and sequencing of the V4 variable region (primers 515F/806R) of the 16S rRNA gene was performed on a MiSeq (Illumina) at a sequencing facility [MR DNA (Molecular Research LP), Shallowater, TX, USA]. The raw sequences were analyzed using a QIIME pipeline (2018.8). Amplicon sequence variants (ASVs) were assigned using DADA and rarefied to 35,000 sequences per sample. Genomic DNA sequences were deposited in a public repository (8).

Extracted DNA was used to perform quantitative PCR for bacterial groups (total bacteria, *Faecalibacterium* spp., *Turicibacter* spp., *Streptococcus* spp., *Escherichia coli, Blautia* spp., *Fusobacterium* spp., *Clostridium hiranonis*) previously associated with dysbiosis and for calculation of the dysbiosis index (9). For these analyses, 2 μl of normalized DNA (final concentration: 5 ng/μl) was combined with 5 μl of a DNA-binding dye (SsoFast EvaGreen® supermix, Catalog #1725201-1725205, Bio-Rad Laboratories, Hercules, CA, USA), 0.4 μl each of a forward and reverse primer (final concentration: 400 nM), and 2.6 μl of PCR water to achieve a total reaction volume of 10 μl. Data were expressed as log amount of DNA (fg) for each particular bacterial group per 10 ng of isolated total DNA.

### Fecal Metabolomics

Fecal metabolomic analysis was performed as per a previous study (4). Briefly, a metabolomics facility (West Coast Metabolomics Core, University of California, Davis, CA, USA) analyzed 10 mg of lyophilized feces from each pooled sample using gas chromatography time-of-flight mass spectrometry and standardized protocols. ChromaTOF v. 2.32 was used to process raw data. BinBase algorithm was applied to match spectra to database compounds or characterize spectra as an unknown compound, and quantification was reported by peak height of an ion at the specific retention index characteristic of the compound across all samples. Metabolomics data were deposited in a public repository (10).

### Statistical Analyses

Descriptive statistics were generated for each response measure. Samples were analyzed for normality using the Shapiro-Wilk test and for the presence of outliers using box-and-whisker plots. A dysbiosis index was calculated. Quantitative Insights Into Microbial Ecology (QIIME) scripts were used to create alpha rarefaction plots, as well as calculate measures of alpha diversity (Chao1, Shannon, Pielou Evenness, and ASVs). Beta diversity was determined using weighted and unweighted Unifrac distance metrics; principal coordinates analysis (PCoA) plots were plotted. Beta diversity across time and groups of dogs was determined using ANOSIM. Global changes in untargeted metabolomic profiles were determined using principal component analysis (PCA) plots and heatmaps. Metabolomic analysis was performed using the *Homo sapiens* pathway library, interquantile range data filtering, log transformation, and Pareto scaling.

Alpha diversity metrics, the dysbiosis index, relative and absolute bacterial abundances, and fecal metabolite profiles were compared between treatment groups using split plot repeated measures mixed models ANOVAs that included fixed effects of treatment group (placebo or synbiotic), time period, and treatment group-by-time period interaction. The repeated measure of time period was accounted for in a repeated statement and random effects for dog nested within group were included. The Shapiro-Wilk test of normality of the residuals was evaluated for each marker to determine if assumption of normally distributed residuals had been met. Model assumptions regarding equality of variances were evaluated with the Levene's test for equality of variances. Box plots and studentized residuals were computed for each model to identify potential outliers. *Post-hoc* differences in least squares means were determined for markers with significant main effects or interaction terms. A rank transformation was applied to qPCR results and relative abundances of ASVs to provide a robust solution to deviations from statistical assumptions. Only ASVs that were present in ≥50% of dogs in ≥1 group at ≥1 time point were included in statistical analyses.

*P* < 0.05 was considered statistically significant. *P*-values were corrected for multiple comparisons on each phylogenetic level and for untargeted metabolomics using the Benjamini and Hochberg's False Discovery Rate (fdr). Publicly-accessible and commercially available software packages were used for all analyses: QIIME 2. Available at: http://www.qiime.org; MetaboAnalyst 4.0. Available at: http://www.metaboanalyst.ca; PRIMER 6, PRIMER-E Ltd; and SAS 9.4 release TS1M3, SAS Institute Inc.

## Results

### Fecal Microbiome

**Figure 2:**
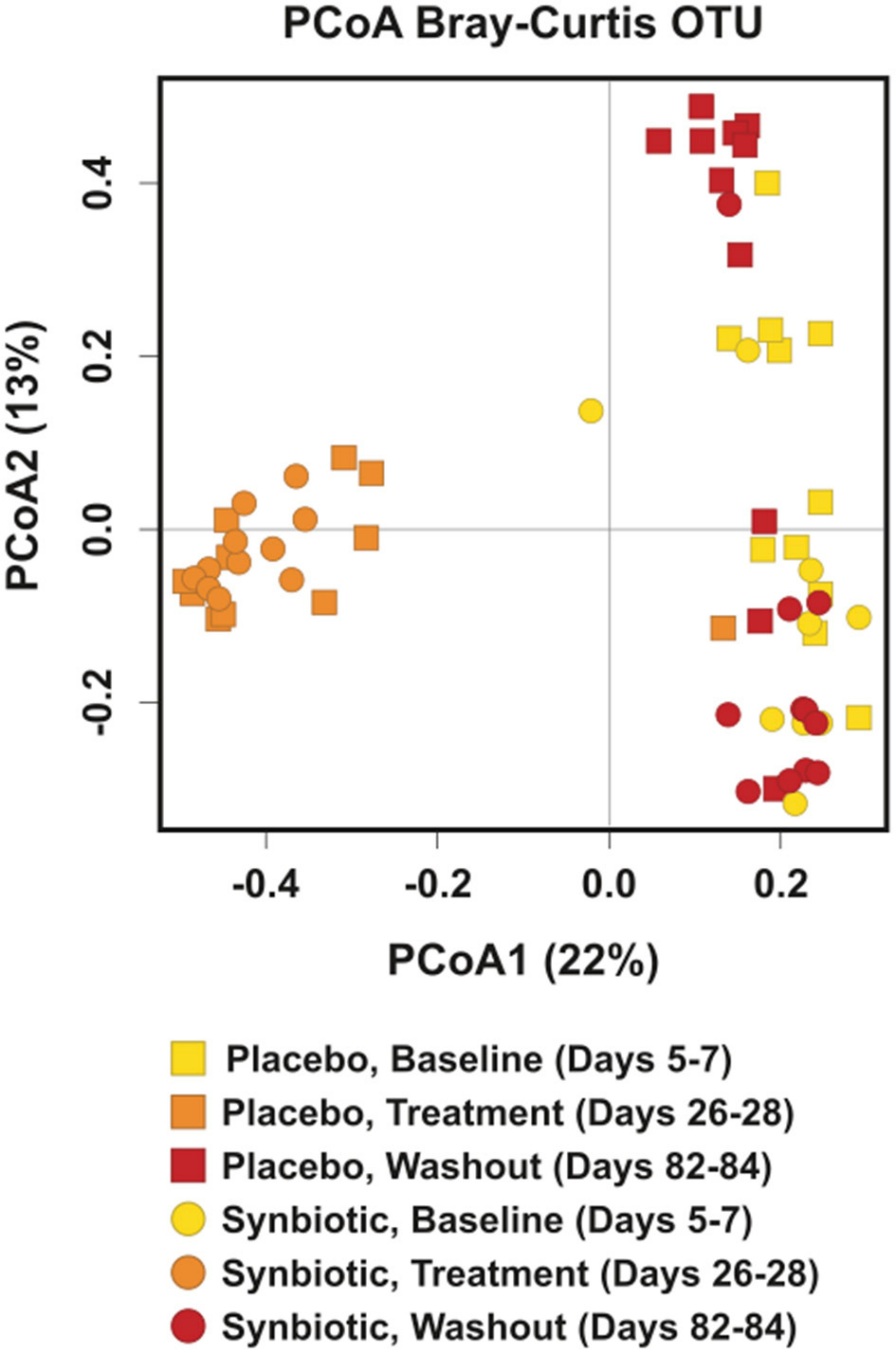
Principal Coordinate Analysis (PCoA) of unweighted UniFrac distances of 16S rRNA genes for dogs that received enrofloxacin/metronidazole followed by placebo or synbiotic for 21 days. Gene sequences were determined using fecal samples collected at the conclusion of baseline (days 5–7), antibiotic administration (days 26–28), and a 56-day washout (days 82–84) from 22 healthy dogs, 11 per group,^+^ that received enrofloxacin (10 mg/kg qd) and metronidazole (12.5 mg/kg BID), followed 1 h later by placebo or a synbiotic combination (BID) PO for 21 days. ^+^Feces from one dog (placebo group) unavailable at the days 26–28 time point.

Alpha diversity differed significantly over time regardless of treatment group, with incomplete recovery of the Shannon index and Chao1 metric on days 82–84 (Table 1). *Post-hoc* analyses revealed all time points differed from one another for both the Shannon index and Chao1 metrics, respectively (*P* ≤ 0.001, for all). A group-by-time interaction was also present for pielou evenness; *post-hoc* analysis revealed this was due to significant differences between treatment groups at the conclusion of treatment (days 26–28) (*P* = 0.023). Beta diversity differed significantly over time and between treatment groups based on unweighted (Figure 2, *P* = 0.001, *R* = 0.516) and weighted UniFrac distances (*P* = 0.001, *R* = 0.609). Based on unweighted distances, beta diversity was significantly different on days 26–28 compared to baseline (*P* = 0.001, *R* = 0.978) and days 82–84 (*P* = 0.001, *R* = 0.923) for dogs in the synbiotic group, with no significant difference between baseline and days 82–84. Conversely, beta diversity was significantly different among all three time points for dogs in the placebo group (*P* = 0.001, *R* = 0.482–0.620). Finally, beta diversity differed slightly between treatment groups at baseline and on days 26–28 (*P* = 0.019–0.023, *R* = 0.130–0.132), whereas a more substantial difference was identified between treatment groups on days 82–84 (*P* = 0.001, *R* = 0.467).

**Table 1:** Mean ± standard deviation (SD) results for alpha diversity metrics collected at the conclusion of baseline (days 5–7), antibiotic administration (days 26–28), and a 56-day washout (days 82–84) from 22 healthy dogs, 11 per group,^+^ that received enrofloxacin (10 mg/kg qd) and metronidazole (12.5 mg/kg BID), followed 1 h later by placebo or a synbiotic combination (BID) PO for 21 days.

	**Baseline**		**Days 26–28**		**Days 82–84**		***P*-value**
	**Placebo**	**Synbiotic**	**Placebo**	**Synbiotic**	**Placebo**	**Synbiotic**	
Shannon index	6.0 ± 0.2^a^	5.7 ± 0.4^a^	5.4 ± 0.2^c^	5.4 ± 0.2^c^	5.7 ± 0.1^b^	5.7 ± 0.2^b^	<0.001 (time)
Pielou evenness	0.9422 ± 0.0082^b^	0.9395 ± 0.0138^b^	0.9452 ± 0.0064^b^	0.9541 ± 0.0081^a^	0.9461 ± 0.0071^b^	0.9427 ± 0.0077^b^	0.007 (time), 0.043 (group by time)
Chao1 metric	80.0 ± 9.1^a^	69.4 ± 14.7^a^	52.5 ± 8.9^c^	50.9 ± 5.7^c^	63.5 ± 5.0^b^	64.3 ± 8.0^b^	<0.001 (time)

+ *Feces from one dog (placebo group) unavailable at the days 26–28 time point. Cells that do not share a common superscript letter differed significantly (P < 0.05) based on post-hoc analysis*.

Five phyla (Table 2) were identified based on sequencing analysis (mean baseline prevalences): *Actinobacteria* (50.54%), *Bacteroidetes* (2.44%), *Firmicutes* (46.12%), *Fusobacteria* (0.04%), and *Proteobacteria* (0.86%). Marked and significant differences were identified among time points and between treatment groups in the fecal microbiome at all phylogenetic levels (Table 2, Supplementary Table 1). At the genus level (Table 2), significant group-by-time, time, and/or treatment group effects were noted for 33 ASVs. Group-by-time interactions were identified for 15 ASVs, whereas concurrent treatment group and time effects—but not group-by-time interactions—were present for six ASVs. Significant time effects were identified for 29 ASVs, 11 of which had neither treatment group nor group-by-time effects.

**Table 2:** Median (range) percent relative abundances of bacterial phyla and genera for dogs that received enrofloxacin/metronidazole followed by placebo or synbiotic for 21 days.

	**Placebo**			**Synbiotic**			**fdr** ***P*****-value**		
	**Baseline**	**Days 26–28**	**Days 82–84**	**Baseline**	**Days 26–28**	**Days 82–84**	Group^*^ Time	**Group**	**Time**
***Actinobacteria***	1.95^c^ (0.59–4.79)	8.22^a^ (2.35–19.04)	6.81^b^ (1.35–11.46)	2.71^c^ (0.47–6.75)	17.23^a^ (2.76–20.19)	4.53^b^ (0.69–13.98)			<0.01
*Bifidobacterium*	0.51^bc^ (0–2.74)	8.22^a^ (0–19.04)	1.57^b^ (0–5.46)	0^d^ (0–1.72)	15.65^a^ (2.61–17.8)	0.58^cd^ (0–4.75)	0.01		<0.01
*Coriobacteriaceae:g_*	0^b^ (0–0.82)	0^b^ (0–0)	1.31^a^ (0–5.57)	0^b^ (0–0)	0^b^ (0–0)	0^b^ (0–8.58)	<0.01	<0.01	<0.01
*Adlercreutzia*	0.19^ab^ (0–0.55)	0^c^ (0–0)	0^b^ (0–0.73)	0^bc^ (0–0.55)	0^c^ (0–0)	0.36^a^ (0–1.29)	0.03		<0.01
*Collinsella*	1.06^a, β^ (0.4–2.62)	0.51^b, β^ (0–2.38)	0.58^a, β^ (0–6.86)	1.79^a, α^ (0.47–6.75)	1.34^b, α^ (0–2.9)	3.25^a, α^ (0.33–6.81)		0.01	0.04
*Slackia*	0^bc^ (0–0.13)	0^c^ (0–0)	0^c^ (0–0)	0^b^ (0–0.3)	0^c^ (0–0)	0.24^a^ (0–0.4)	0.01	0.03	0.01
***Bacteroidetes***	18.63^a, α^ (6.36–30.48)	0.22^c, α^ (0–10.9)	20.18^b, α^ (0.97–31.88)	19.11^a, β^ (0.43–29.42)	0^c, β^ (0–0.18)	3.41^b, β^ (0–9.27)		0.01	<0.01
*Bacteroides*	5.11^a^ (0.61–12.81)	0^c^ (0–6.54)	6.8^b^ (0–13.1)	6.12^a^ (0–17.99)	0^c^ (0–0.18)	1.65^b^ (0–6.67)			<0.01
*Prevotella*	0.27^a, α^ (0–1.34)	0^c, α^ (0–0)	0^b, α^ (0–3.39)	0.22^a, β^ (0–0.64)	0^c, β^ (0–0.1)	0^b, β^ (0–1.21)		<0.01	<0.01
*S24_7:g_*	1.5^ab^ (0–9.73)	0^d^ (0–0.67)	8.39^a^ (0–15.2)	0.16^bc^ (0–8.82)	0^d^ (0–0)	0^cd^ (0–7.05)	0.02	<0.01	<0.01
*[Paraprevotellaceae]:g_*	0.27^a^ (0–1.34)	0^b^ (0–0)	0^a^ (0–3.39)	0.22^a^ (0–0.64)	0^b^ (0–0.1)	0^a^ (0–1.21)			<0.01
*[Prevotella]*	6.73^a, α^ (1.97–13.7)	0^c, α^ (0–4.4)	1.67^b, α^ (0–7.27)	2.67^a, β^ (0.12–12.22)	0^c, β^ (0–0)	0^b, β^ (0–2.94)		<0.01	<0.01
***Firmicutes***	64.62^b^ (46.15–81.31)	88.53^a^ (71.55–94.76)	66.8^b^ (49.2–87.16)	57.75^b^ (44.63–92.6)	82.77^a^ (79.81–96.84)	79.11^a^ (70.93–94.58)	0.04		<0.01
*Enterococcus*	0^b^ (0–0)	0^a^ (0–5.17)	0^b^ (0–0)	0^b^ (0–0)	1.71^a^ (0–3.75)	0^b^ (0–0)			<0.01
*Lactobacillus*	0.48^b^ (0–11.24)	32.7^a^ (0–54.52)	0.4^b^ (0–1.65)	0.5^b^ (0–23.85)	25.96^a^ (18.31–50.35)	0.41^b^ (0–7.56)			<0.01
*Streptococcus*	0.19^c^ (0–1.48)	26.89^a^ (0–41.4)	0.4^b^ (0–6.88)	0^c^ (0–4.83)	18.42^a^ (3.59–36.64)	5.31^b^ (0–17.5)			<0.01
*Turicibacter*	5.9^ab^ (1.38–13.21)	0^d^ (0–0.62)	2.18^c^ (0–8.86)	3.25^bc^ (0–19.05)	0^d^ (0–0.27)	9.43^a^ (1.16–25.61)	<0.01		<0.01
*Clostridiaceae:_*	7.4^ab^ (5.66–14.95)	0.4^c^ (0–0.98)	2.73^b^ (0.91–17.52)	9.68^a^ (0.58–18.58)	0^c^ (0–0.4)	11.99^a^ (5.97–16.48)	0.01		<0.01
*Clostridiaceae:g_*	0.29^a^ (0–1.91)	0^b^ (0–5.08)	0.64^a^ (0–2.78)	1.49^a^ (0–3.02)	0^b^ (0–1.07)	1.63^a^ (0–3.04)			0.01
*Clostridium*	0^c^ (0–0.14)	0^c^ (0–0.7)	0^bc^ (0–2.08)	0^ab^ (0–3.61)	0^c^ (0–0)	0.57^a^ (0–10.36)	0.05		<0.01
*Lachnospiraceae:_*	3.78^bc^ (1–5.6)	5.65^a^ (2.1–13.67)	1.68^c^ (0–9.23)	3.2^bc^ (1.29–8.72)	4.57^ab^ (1.07–16.09)	5.88^a^ (3.42–9.01)	0.01		
*Blautia*	3.47^β^ (0.54–8.09)	2.93^β^ (0–16.39)	3.12^β^ (0–15.33)	9.36^α^ (0–18.48)	3.98^α^ (0.5–11.03)	10.31^α^ (2.03–15.09)		0.03	
*[Ruminococcus]*	2.68^b^ (0–4.8)	3.5^ab^ (0–14.16)	5.07^a^ (0–13.45)	5.08^a^ (2.16–8.94)	2.47^ab^ (0–19.82)	4.67^a^ (2.26–8.26)	0.05		
*Peptococcus*	0.53^a^ (0–1.28)	0^b^ (0–0.11)	0^b^ (0–1.39)	0.38^a^ (0–2.33)	0^b^ (0–0)	0.92^a^ (0–2.38)	0.01		<0.01
*Peptostreptococcus*	0^b, α^ (0–0.28)	0^b, α^ (0–0.3)	0.96^a, α^ (0–6.82)	0^b, β^ (0–0)	0^b, β^ (0–0)	0^a, β^ (0–7.59)		0.02	<0.01
*Ruminococcaceae:g_*	1.34^a, α^ (0–2.74)	0^b, α^ (0–0.87)	0.57^a, α^ (0.22–1.27)	0.23^a, β^ (0–1.83)	0^b, β^ (0–0)	0.27^a, β^ (0–2.02)		0.03	<0.01
*Faecalibacterium*	3.12^a^ (0.53–8.38)	0^c^ (0–1.32)	0^b^ (0–2.18)	2.44^a^ (0–10.02)	0^c^ (0–0.11)	0.94^b^ (0–5.1)			<0.01
*Megamonus*	1.05^a^ (0–3.72)	0^c^ (0–0)	0^b^ (0–1.96)	0.76^a^ (0–4.64)	0^c^ (0–0.24)	0.37^b^ (0–1.64)			<0.01
*Phascolarctobacterium*	1.51^a^ (0.4–2.75)	0^c^ (0–0.59)	0.07^b^ (0–1.21)	0.64^a^ (0–2.69)	0^c^ (0–0)	0^b^ (0–1.01)			<0.01
*Erysipelotrichaceae:g_*	0.48^ab^ (0.11–1.79)	0^cd^ (0–1.39)	0.18^bc^ (0–3.06)	1.02^a^ (0–3.11)	0^d^ (0–0)	1.21^a^ (0–4.22)	0.01		<0.01
*Allobaculum*	19.85^b^ (0.56–51.64)	5.39^cd^ (1.88–21.72)	39.42^a^ (4.07–49.3)	4.2^d^ (0–29.34)	11.2^bc^ (0–22.04)	2.09^d^ (1.12–40.59)	<0.01	0.02	
*Catenibacterium*	0.67^a, β^ (0–8.1)	0^b, β^ (0–4.84)	0^a, β^ (0–11.62)	6.17^a, α^ (0–12.65)	0.23^b, α^ (0–2.32)	4.48^a, α^ (0–16.53)		0.01	<0.01
*[Eubacterium]*	0.89^a^ (0.4–5.16)	0.07^b^ (0–10.31)	0^b^ (0–9.22)	4.88^a^ (0.5–6.45)	0^b^ (0–0.27)	4.93^a^ (0–10.86)	<0.01	0.03	<0.01
***Fusobacteria***	12^a^ (6.39–18.91)	0^c^ (0–14.44)	5.57^b^ (1.38–11.9)	12^a^ (0–27.67)	0^c^ (0–0.3)	9.26^b^ (0–17.19)			<0.01
*Fusobacterium*	12^a^ (6.39–18.91)	0^c^ (0–14.44)	5.57^b^ (1.38–11.9)	11.96^a^ (0–21.48)	0^c^ (0–0.3)	9.26^b^ (0–17.19)			<0.01
***Proteobacteria***	2.2^a^ (0.63–4.59)	0.76^b^ (0–4.51)	1.61^ab^ (0–11.4)	2.89^a^ (0.4–7.67)	0.17^b^ (0–6.85)	1.68^ab^ (0–3.72)			0.01
*Succinivibrionaceae:g_*	0.66^a^ (0.25–1.96)	0^b^ (0–0)	0^b^ (0–1)	0.82^a^ (0–4.06)	0^b^ (0–0)	0.69^a^ (0–2.78)	0.04		<0.01

*P-values were adjusted based on the Benjamini and Hochberg False discovery rate (fdr). Relative abundances that do not share a common superscript letter differed significantly (fdr-adjusted P < 0.05) based on post-hoc analysis*.

**Figure 3:**
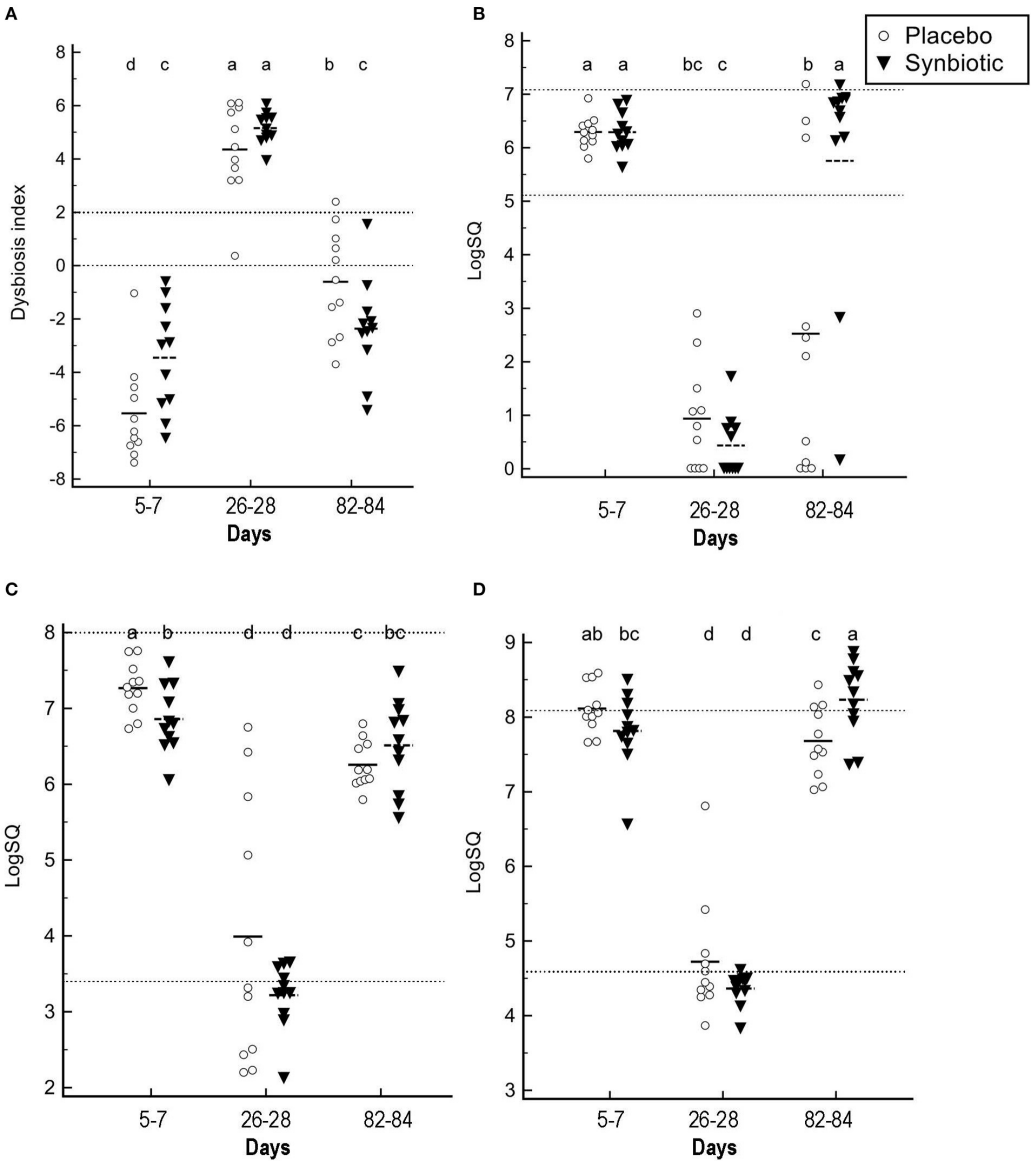
Fecal dysbiosis index results and selected bacterial abundances determined by qPCR. **(A)** Dysbiosis index. **(B)** *C. hiranonis*. **(C)** *Faecalibacterium*. **(D)** *Turicibacter*. Medians and individual values are presented for dogs in the placebo (open circles) and synbiotic (black triangles) treatment groups. Dotted lines indicate the reference interval. For the dysbiosis index, values <0 indicate normobiosis, between 0 and 2 are equivocal, and >2 indicate dysbiosis. Significance was set as *P* < 0.05. Abundances that do not share a letter differed significantly based on *post-hoc* analysis.

With regard to quantitative PCR results, group-by-time interactions and time effects were identified for the dysbiosis index and abundances for several bacteria (Figure 3). Significant group-by-time interactions were identified for *Faecalibacterium* (*P* = 0.02), *Turicibacter* (*P* = 0.002), *Streptococcus* (*P* = 0.048), *Blautia* (*P* = 0.042), and *C. hiranonis* (*P* < 0.001). Main effect differences over time were observed for *E. coli, Fusobacterium*, and total bacteria (*P* < 0.001, for each). Dysbiosis index values differed over time based on treatment received (*P* = 0.002). *Post-hoc* tests revealed that the dysbiosis index was significantly higher on days 26–28 when compared to days 5–7 for both treatment groups (*P* < 0.001, for each), with return to baseline values at days 82–84 in dogs administered the synbiotic but not the placebo. This difference primarily was due to return of *C. hiranonis*, as well as *Faecalibacterium* and *Turicibacter*, abundances to baseline values for dogs in the synbiotic group (Figure 3). Total abundance of bacteria at days 82–84 did not differ from baseline for either group.

### Fecal Metabolomics

**Figure 4:**
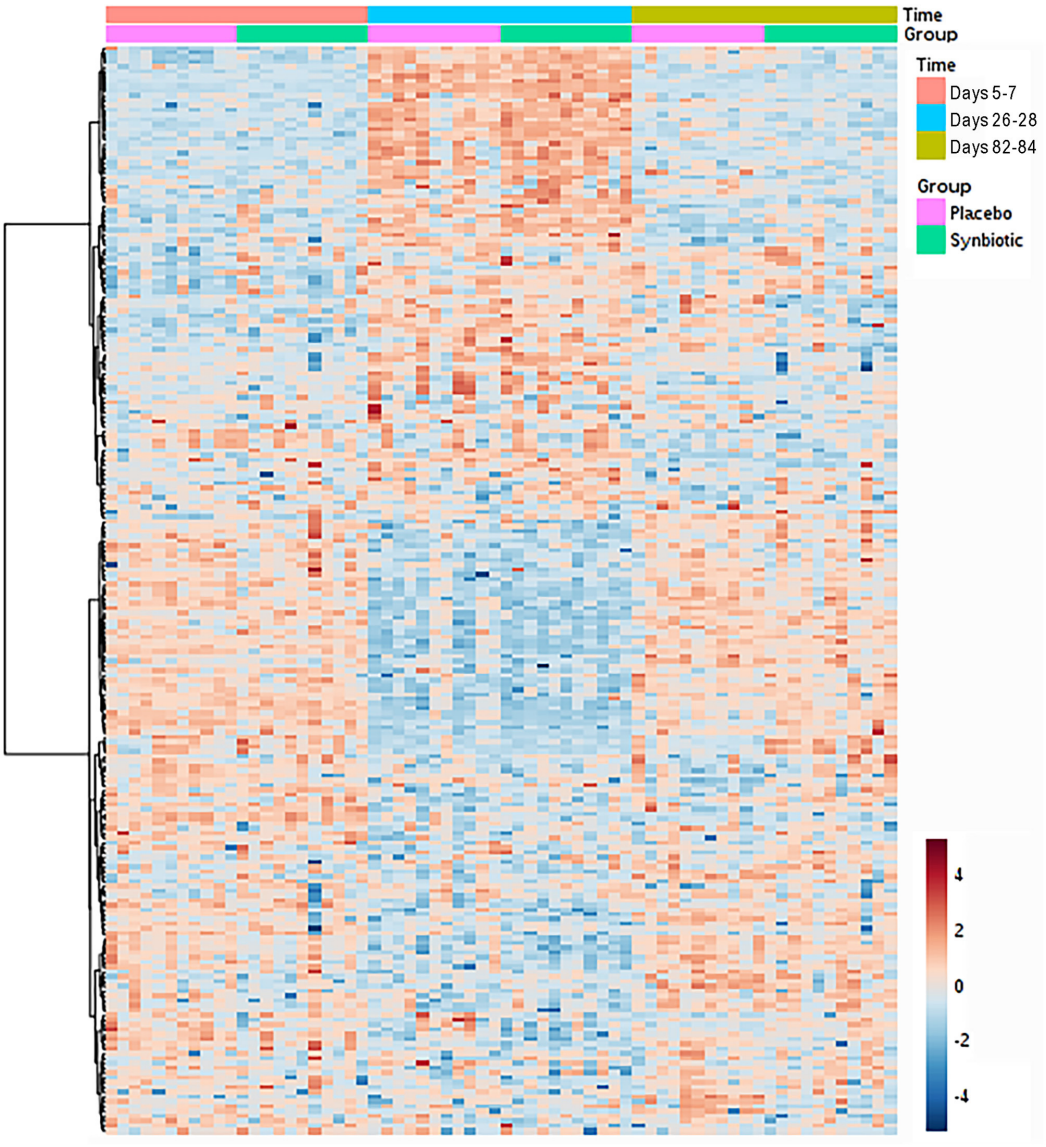
Dual hierarchical dendrogram of fecal metabolites, clustered by pathway, that differed significantly over time for dogs that received enrofloxacin/metronidazole followed by placebo or synbiotic for 21 days.

Based on comparison of spectral analysis results to database compounds, 227 compounds were identified. Profiles for 196 metabolites differed significantly (fdr-adjusted *P* < 0.05) over the course of the study (Figure 4, Table 3, Supplementary Table 2). Treatment group-by-time interactions were present for 28 metabolites, whereas concurrent group and time effects were identified for 20 metabolites. One hundred fifty-one metabolites had significant temporal changes alone or in combination with group-by-time interactions.

**Table 3:** Median (range) peak height of fecal metabolites with significant group-by-time interactions or concurrent group and time effects for dogs that received enrofloxacin/metronidazole followed by placebo or synbiotic for 21 days.

	**Placebo**			**Synbiotic**			**fdr** ***P*****-value**		
	**Baseline**	**Days 26–28**	**Days 82–84**	**Baseline**	**Days 26–28**	**Days 82–84**	Group* Time	**Group**	**Time**
1-deoxyerythritol	3,943^a,β^ (2,376–6,884)	1,759^c,β^ (1,115–3,795)	2,573^b,β^ (1,251–10,686)	5,810^a,α^ (3,564–10,619)	3,286^c,α^ (1,783–4,037)	3,514^b,α^ (2,194–5,834)		<0.01	<0.01
2-hydroxyglutaric acid	617^bc^ (351–1,769)	752^b^ (269–8,379)	961^b^ (242–3,611)	497^cd^ (264–881)	2,999^a^ (734–10,872)	336^d^ (185–733)	<0.01		<0.01
2-monoolein	13,913^a^ (9,341–31,985)	4,732^c^ (2,417–9,397)	10,091^b^ (4,217–15,122)	10,370^b^ (996–23,452)	4,657^c^ (2,260–9,157)	14,264^ab^ (5,223–17,870)	0.02		<0.01
3-phenyllactic acid	1,589^d^ (621–6,453)	20,424^a^ (13,289–58,071)	12,004^b^ (2,943–26,814)	2,804^cd^ (1,659–7,204)	35,635^a^ (25,628–54,631)	5,172^c^ (1,561–10,187)	0.02		<0.01
Allantoic acid	574^ab^ (346–9,428)	363^ab^ (57–5,302)	372^b^ (98–1,417)	479^b^ (262–7,659)	525^b^ (71–8,130)	1,060^a^ (237–8,365)	0.045		
Behenic acid	17,039^a^ (10,554–27,314)	12,039^b^ (5,630–33,623)	8,485^c^ (6,598–16,857)	18,397^a^ (5,337–37,951)	8,169^c^ (4,824–16,919)	9,831^bc^ (3,974–17,712)	0.045		<0.01
Butane-2,3-diol NIST	3,568^c^ (2,699–7,200)	66,839^ab^ (1,848–177,389)	14,081^ab^ (1,978–946,157)	5,832^bc^ (2,181–74,843)	73,860^a^ (4,516–207,742)	3,401^c^ (831–20,343)	<0.01		<0.01
Cholesterol	149,365^a,α^ (103,904–195,152)	75,299^c,α^ (32,531–146,078)	83,947^b,α^ (55,654–152,213)	100,350^a,β^ (75,261–321,230)	52,181^c,β^ (37,103–73,759)	87,172^b,β^ (51,653–178,799)		0.04	<0.01
Daidzein	473^b,β^ (251–5,277)	9,026^a,β^ (2,351–11,387)	368^b,β^ (217–9,333)	921^b,α^ (448–15,926)	8,394^a,α^ (1,659–15,777)	5,369^b,α^ (397–15,651)		0.01	<0.01
Deoxycholic acid	420,927^a^ (287,558–1,547,882)	2,962^cd^ (1,273–413,873)	149,001^c^ (872–267,037)	225,249^b^ (110,916–674,640)	2,284^d^ (733–6,615)	255,829^b^ (3,037–756,630)	0.02		<0.01
D-erythro-sphingosine	6,805^a^ (2,526–29,549)	3,756^bc^ (975–18,571)	2,036^cd^ (915–5,138)	4,890^ab^ (955–11,023)	1,622^d^ (672–9,162)	4,518^ab^ (3,092–14,801)	0.01		<0.01
Docosahexaenoic acid	17,299^a,α^ (13,147–48,544)	5,222^c,α^ (988–12,968)	16,575^b,α^ (7,393–31,581)	11,549^a,β^ (6,307–34,081)	1,527^c,β^ (1,433–5,486)	10,141^b,β^ (2,105–18,520)		0.02	<0.01
Erythritol	4,250^b,β^ (2,176–21,769)	13,219^a,β^ (1,480–31,839)	913^c,β^ (413–11,712)	4,025^b,α^ (907–19,062)	24,525^a,α^ (20,722–46,236)	2,312^c,α^ (852–10,345)		0.01	<0.01
Ferulic acid	1,192^bc^ (652–1,736)	1,920^ab^ (408–4,344)	563^d^ (137–1,194)	861^cd^ (405–1,233)	2,122^a^ (1,300–3,853)	964^bc^ (522–2,479)	0.03		<0.01
Fucose	179,811^a^ (41,782–233,563)	137,082^a^ (3,849–837,808)	13,332^c^ (8,185–320,277)	137,859^ab^ (50,951–233,808)	68,870^bc^ (1,751–169,428)	120,553^ab^ (17,516–264,172)	0.01		
Galactinol	695^b,β^ (534–1,667)	981^a,β^ (823–2,975)	522^c,β^ (303–1,023)	1,050^b,α^ (518–2,174)	1,893^a,α^ (1,219–3,985)	500^c,α^ (279–1,951)		0.047	<0.01
Galactose	163,560^a^ (56,259–288,199)	39,870^bc^ (19,078–237,206)	24,910^cd^ (8,818–308,074)	102,098^ab^ (46,025–202,159)	27,749^d^ (7,301–69,499)	121,530^ab^ (28,291–493,825)	0.03		<0.01
Glutamic acid	121,446^b^ (95,863–179,885)	250,811^a^ (116,078–408,091)	132,799^b^ (60,403–435,886)	135,038^b^ (95,213–274,191)	275,893^a^ (162,620–470,665)	80,460^c^ (30,579–124,299)	0.01		<0.01
Glutaric acid	896^a,β^ (426–1,641)	768^a,β^ (318–3,303)	470^b,β^ (303–1,245)	1,141^a,α^ (554–2,124)	1,343^a,α^ (530–8,514)	573^b,α^ (519–1,390)		0.03	<0.01
Glycerol-alpha-phosphate	1,332^c^ (130–2,070)	2,743^b^ (774–4,096)	1,420^bc^ (774–2,365)	1,055^c^ (95–2,587)	3,960^a^ (2,865–5,321)	1,224^c^ (253–1,980)	0.03		<0.01
Hexadecylglycerol NIST	6,021^a,α^ (4,332–11,689)	2,783^c,α^ (620–3,860)	4,273^b,α^ (1,897–5,447)	3,592^a,β^ (2,727–12,350)	1,632^c,β^ (678–3,580)	2,959^b,β^ (1,246–5,882)		0.02	<0.01
Indole-3-propionic acid	14,629^a,α^ (8,342–41,807)	6,359^b,α^ (273–11,067)	21,609^a,α^ (11,397–213,321)	9,929^a,β^ (6,433–39,621)	4,103^b,β^ (122–8,132)	9,006^a,β^ (5,149–261,311)		0.01	<0.01
Inosine	843^a^ (191–18,392)	178^c^ (103–4,221)	437^b^ (134–6,505)	2,812^a^ (475–16,194)	145^c^ (96–253)	6,424^a^ (417–12,002)	0.01		<0.01
Isoleucine	218,417^c^ (144,351–405,106)	382,705^ab^ (274,560–647,973)	442,249^a^ (270,033–602,789)	295,250^ab^ (214,178–674,190)	322,186^b^ (183,901–691,552)	420,279^a^ (250,849–929,432)	0.02		<0.01
Linoleic acid	31,003^a,α^ (19,842–58,595)	6,812^b,α^ (2,085–17,820)	25,853^a,α^ (12,932–47,171)	14,912^a,β^ (8,022–36,173)	4,846^b,β^ (1,882–8,382)	25,814^a,β^ (11,785–33,998)		0.02	<0.01
Lysine	91,686^a,α^ (59,571–204,631)	37,443^b,α^ (4,793–129,197)	195,379^a,α^ (4,376–244,884)	88,520^a,β^ (35,630–172,381)	37,148^b,β^ (19,483–63,945)	75,462^a,β^ (8,834–135,588)		0.04	<0.01
Maltose	3,321^ab^ (1,174–15,494)	2,055^b^ (443–12,081)	5,510^a^ (990–13,210)	2,142^b^ (1,296–12,555)	4,835^a^ (1,370–26,980)	2,483^b^ (1,114–6,542)	0.02		
Methionine sulfoxide	25,019^b,α^ (16,164–32,096)	15,187^c,α^ (3,893–40,207)	43,425^a,α^ (24,804–61,200)	27,844^b,β^ (9,120–42,782)	10,510^c,β^ (5,714–17,172)	29,577^a,β^ (7,316–54,293)		0.02	<0.01
N-acetylglutamate	611^a^ (452–1,813)	545^a^ (306–1,779)	856^a^ (251–3,533)	585^a^ (418–2,128)	570^a^ (269–2,982)	253^b^ (189–1,387)	0.02		
Ornithine	39,943^b,α^ (7,429–60,274)	32,926^b,α^ (3,931–67,775)	106,782^a,α^ (37,067–161,661)	41,914^b,β^ (14,623–55,508)	31,069^b,β^ (13,064–44,642)	38,525^a,β^ (14,556–67,033)		<0.01	0.01
Oxoproline	61,545^bc^ (54,924–112,476)	340,035^a^ (82,079–490,827)	91,480^b^ (48,067–308,646)	75,373^bc^ (48,333–228,173)	390,081^a^ (316,439–630,055)	50,243^c^ (38,250–137,407)	0.03		<0.01
Phenylacetic acid	4,975^b^ (3,312–24,386)	1,324^c^ (446–6,138)	9,494^ab^ (3,106–20,887)	11,517^a^ (3,607–50,106)	908^c^ (626–3,941)	9,363^ab^ (2,780–23,596)	0.04		<0.01
P-hydroxylphenyllactic acid	552^c^ (305–1,332)	2,802^a^ (990–7,942)	2,157^b^ (747–5,019)	645^c^ (236–3,049)	5,232^a^ (1,761–8,091)	856^c^ (402–1,859)	0.01		<0.01
Phytosphingosine	6,552^a^ (4,474–14,350)	3,373^bc^ (1,053–36,357)	5,011^bc^ (2,568–5,546)	4,056^c^ (1,714–8,857)	2,686^d^ (1,639–6,759)	5,229^ab^ (3,312–11,865)	0.02		<0.01
Resorcinol	1,122^a^ (263–1,440)	86^c^ (60–144)	298^b^ (127–1,160)	417^b^ (147–2,294)	89^c^ (67–107)	543^b^ (121–1,962)	0.03		<0.01
Ribonic acid	1,295^bc^ (841–2,265)	2,623^a^ (1,690–6,569)	1,252^b^ (659–2,982)	1,182^bc^ (412–1,987)	3,303^a^ (2,363–4,531)	961^c^ (509–1,395)	0.03		<0.01
Sinapinic acid	793^b,β^ (195–1,046)	1,872^a,β^ (358–4,872)	593^b,β^ (459–776)	776^b,α^ (561–1,769)	2,750^a,α^ (1,008–5,776)	782^b,α^ (531–1,458)		0.02	<0.01
Sorbitol	7,753^a,β^ (1,676–58,914)	5,530^a,β^ (1,395–51,870)	1,853^b,β^ (1,049–2,261)	5,343^a,α^ (2,302–44,389)	9,181^a,α^ (3,005–54,449)	3,107^b,α^ (656–7,939)		0.02	<0.01
Thymidine	9,507^a,α^ (4,687–14,694)	2,350^c,α^ (1,095–7,641)	7,075^b,α^ (1,920–11,952)	7,150^a,β^ (4,227–11,471)	1,826^c,β^ (823–3,809)	4,999^b,β^ (1,774–12,346)		0.046	<0.01
Thymine	46,343^b^ (27,601–68,823)	8,471^d^ (3,608–26,848)	30,637^c^ (17,424–50,012)	68,273^a^ (43,452–77,166)	6,774^d^ (4,152–15,770)	45,188^bc^ (24,105–75,344)	0.04		<0.01
Tocopherol acetate	2,491^a,β^ (1,105–7,290)	1,728^b,β^ (379–3,256)	1,764^b,β^ (817–5,111)	5,993^a,α^ (1,472–8,182)	2,087^b,α^ (934–3,937)	3,653^b,α^ (760–10,497)		0.03	<0.01
Trans-4-hydroxyproline	9,801^b,β^ (5,163–14,095)	12,876^a,β^ (6,879–29,372)	10,814^b,β^ (6,362–22,934)	14,413^b,α^ (6,857–26,266)	22,303^a,α^ (15,712–34,354)	12,084^b,α^ (8,242–21,617)		0.01	<0.01
Trehalose	818^c^ (267–39,653)	3,345^b^ (424–15,841)	1,446^bc^ (355–4,849)	1,515^bc^ (469–33,179)	24,151^a^ (949–67,966)	612^c^ (289–5,888)	0.04		<0.01
Tyramine	144,932^cd^ (44,639–798,913)	465,802^a^ (273,758–710,593)	95,257^d^ (14,855–160,213)	227,211^bc^ (6,229–739,225)	344,927^ab^ (222,062–866,039)	387,329^ab^ (81,658–1,045,046)	0.02		<0.01
Uridine	2,015^a^ (779–6,617)	791^**b**^ (426–3,758)	1,089^b^ (614–4,056)	2,317^a^ (1,511–17,296)	923^b^ (520–1,473)	4,861^a^ (1,110–10,101)	0.01	0.01	<0.01
Urocanic acid	1,247^a,β^ (733–2,876)	1,023^b,β^ (263–3,379)	1,406^a,β^ (598–3,396)	2,186^a,α^ (869–3,699)	1,114^b,α^ (420–2,808)	1,950^a,α^ (1,220–2,390)		0.03	0.03
Xanthosine	494^a^ (204–2,836)	98^c^ (47–641)	195^b^ (60–1,004)	774^a^ (290–2,233)	102^c^ (76–123)	543^a^ (281–1,597)	0.02		<0.01

*Profiles that do not share a common superscript letter differed significantly (fdr-adjusted P < 0.05) based on post-hoc analysis*.

A variety of significant temporal patterns of change were identified. Profiles for 73 metabolites differed from baseline on days 26–28 with return to baseline values at day 82–84, whereas recovery to baseline values was incomplete for an additional 45 metabolites. Twenty metabolites had profiles that differed from baseline on days 26–28 without significant change thereafter. Profiles for 17 metabolites were significantly different at days 82–84 compared to baseline and days 26–28, with no difference between profiles for the other two time points. Finally, 16 metabolites had significant derangements compared to baseline on days 26–28 with overcorrection past baseline values on days 82–84. More complex patterns of change were noted for dogs with treatment group-by-time interactions.

Significant group-by-time, group, and time effects were identified for 35 metabolites related to short-chain fatty acid (SCFA), bile acid, tryptophan, and sphingolipid metabolism (Table 4). Significant associations were also present for profiles related to cinnaminic acid and benzoic acid metabolites. Finally, group-by-time and time effects were noted for profiles for fucose and ethanolamine, respectively.

**Table 4:** Median (range) peak height of metabolites of known biological importance with profiles that significantly differed over the course of the study in feces collected at the conclusion of baseline (days 5–7), antibiotic administration (days 26–28), and a 56-day washout (days 82–84) from 22 healthy dogs, 11 per group,^+^ that received enrofloxacin (10 mg/kg qd) and metronidazole (12.5 mg/kg BID), followed 1 h later by placebo or a synbiotic combination (BID) PO for 21 days.

	**Placebo**			**Synbiotic**			**fdr** ***P*****-value**		
	**Baseline**	**Days 26–28**	**Days 82–84**	**Baseline**	**Days 26–28**	**Days 82–84**	Group^*^ Time	**Group**	**Time**
**Short chain fatty acid metabolites**									
2,4-diaminobutyric acid	5,551^a^ (1,401–9,021)	1,223^c^ (417–12,349)	2,661^b^ (1,021–3,656)	5,124^a^ (1,888–7,023)	1,042^c^ (523–2,201)	2,845^b^ (903–5,453)			<0.01
2-aminobutyric acid	90,013^b^ (60,021–161,873)	20,331^c^ (3,904–96,172)	130,033^a^ (44,028–344,232)	122,729^b^ (36,089–212,264)	8,978^c^ (5,239–62,097)	136,646^a^ (85,394–326,247)			<0.01
2-deoxytetronic acid	4,042^a^ (1,569–22,356)	1,157^b^ (186–14,752)	2,324^a^ (1,540–8,108)	5,048^a^ (1,250–49,806)	892^b^ (526–1,978)	3,829^a^ (1,140–14,386)			<0.01
2-hydroxybutanoic acid	5,994^b^ (3,221–29,404)	12,674^b^ (4,825–22,146)	24,725^a^ (9,275–51,409)	7,342^b^ (3,971–113,729)	9,169^b^ (1,147–37,955)	16,023^a^ (6,796–46,386)			<0.01
2-hydroxyvaleric acid	4,623^a^ (3,406–5,736)	1,476^b^ (540–11,370)	4,689^a^ (1,896–6,488)	4,375^a^ (2,677–21,425)	965^b^ (597–9,128)	4,067^a^ (1,883–8,770)			<0.01
3-aminoisobutyric acid	7,459^a^ (3,499–36,854)	5,247^b^ (210–8,123)	32,758^a^ (845–55,405)	7,710^a^ (3,770–20,700)	3,380^b^ (460–12,971)	2,883^a^ (2,204–78,482)			0.01
3-(3-hydroxyphenyl)propionic acid	261,529^a^ (149,818–410,768)	1,879^c^ (1,325–26,908)	127,896^b^ (1,944–250,573)	129,710^a^ (3,303–569,367)	1,527^c^ (924–4,003)	142,548^b^ (14,129–321,723)			<0.01
3-(4-hydroxyphenyl)propionic acid	89,461^b^ (18,211–355,326)	26,006^c^ (8,474–99,922)	269,044^a^ (45,812–687,935)	121,925^b^ (28,869–268,775)	20,974^c^ (6,288–35,824)	155,582^a^ (86,037–894,720)			<0.01
3-hydroxybutyric acid	7,184^a^ (3,027–10,079)	2,712^b^ (237–11,586)	7,454^a^ (2,366–13,042)	10,391^a^ (3,067–50,349)	1,618^b^ (443–7,224)	4,380^a^ (1,754–46,165)			<0.01
3-phenyllactic acid	1,589^d^ (621–6,453)	20,424^a^ (13,289–58,071)	12,004^b^ (2,943–26,814)	2,804^cd^ (1,659–7,204)	35,635^a^ (25,628–54,631)	5,172^c^ (1,561–10,187)	0.02		<0.01
4-aminobutyric acid	6,834^a^ (5,180–11,170)	1,610^b^ (718–10,973)	2,702^b^ (190–5,965)	5,889^a^ (2,841–20,586)	3,310^b^ (1,330–10,461)	3,585^b^ (1,658–6,878)			<0.01
4-hydroxybutyric acid	1,814^b^ (1,170–10,221)	3,859^a^ (2,692–7,318)	3,519^b^ (1,627–6,761)	1,843^b^ (1,206–4,719)	4,089^a^ (2,598–6,759)	1,972^b^ (1,213–6,025)			<0.01
4-hydroxyphenylacetic acid	42,535^a^ (27,466–79,397)	19,423^b^ (2,564–41,632)	61,252^a^ (21,157–105,898)	41,639^a^ (19,711–123,186)	16,726^b^ (160–24,795)	34,960^a^ (18,054–104,601)			<0.01
Butane-2,3-diol NIST	3,568^c^ (2,699–7,200)	66,839^ab^ (1,848–177,389)	14,081^ab^ (1,978–946,157)	5,832^bc^ (2,181–74,843)	73,860^a^ (4,516–207,742)	3,401^c^ (831–20,343)	<0.01		<0.01
Butyrolactam NIST	6,568^a^ (4,141–17,913)	2,789^c^ (1,935–5,064)	4,346^b^ (2,453–5,128)	5,853^a^ (4,049–11,196)	3,469^c^ (1,678–5,100)	3,629^b^ (2,463–8,860)			<0.01
Lactic acid	580^b^ (187–136,848)	864,555^a^ (184–1,701,564)	36,973^b^ (136–169,063)	524^b^ (174–79,629)	1,322,605^a^ (117–2,202,609)	17,542^b^ (172–125,633)			<0.01
Phenylacetic acid	4,975^b^ (3,312–24,386)	1,324^c^ (446–6,138)	9,494^ab^ (3,106–20,887)	11,517^a^ (3,607–50,106)	908^c^ (626–3,941)	9,363^ab^ (2,780–23,596)	0.04		<0.01
P-hydroxylphenyllactic acid	552^c^ (305–1,332)	2,802^a^ (990–7,942)	2,157^b^ (747–5,019)	645^c^ (236–3,049)	5,232^a^ (1,761–8,091)	856^c^ (402–1,859)	0.01		<0.01
Propane-1,3-diol NIST	6,582^b^ (4,250–10,253)	9,521^a^ (2,735–17,768)	5,215^b^ (2,976–7,953)	4,907^b^ (1,197–11,000)	10,327^a^ (3,009–15,544)	4,317^b^ (531–7,219)			0.01
**Bile acid metabolites**									
Cholesterol	149,365^a, a^ (103,904–195,152)	75,299^c, a^ (32,531–146,078)	83,947^b, a^ (55,654–152,213)	100,350^a, b^ (75,261–321,230)	52,181^c, b^ (37,103–73,759)	87,172^b, b^ (51,653–178,799)		0.04	<0.01
Cholic acid	2,933^c^ (1,456–13,399)	62,504^a^ (1,494–318,640)	9,197^b^ (1,883–71,273)	2,849^c^ (1,221–13,634)	110,785^a^ (43,650–229,826)	6,631^b^ (1,267–95,346)			<0.01
Deoxycholic acid	420,927^a^ (287,558–1,547,882)	2,962^cd^ (1,273–413,873)	149,001^c^ (872–267,037)	225,249^b^ (110,916–674,640)	2,284^d^ (733–6,615)	255,829^b^ (3,037–756,630)	0.02		<0.01
Dihydrocholesterol	1,960^a^ (1,360–2,384)	870^c^ (630–1,538)	1,634^b^ (923–2,355)	1,659^a^ (1,017–4,691)	774^c^ (556–1,369)	1,225^b^ (253–2,462)			<0.01
Lithocholic acid	34,659^a^ (2,094–119,375)	647^c^ (401–31,262)	10,073^b^ (576–24,309)	25,565^a^ (4,269–66,206)	496^c^ (344–810)	18,850^b^ (639–39,383)			<0.01
**Tryptophan metabolites**									
Indole-3-acetate	20,743^a^ (14,077–23,638)	3,828^b^ (1,115–9,503)	22,084^a^ (12,120–48,972)	18,542^a^ (10,247–27,294)	2,643^b^ (1,675–4,983)	18,757^a^ (11,691–99,336)			<0.01
Indole-3-lactate	115,052^a^ (74,011–172,000)	34,761^c^ (16,259–45,655)	48,501^b^ (19,622–126,079)	93,617^a^ (46,576–150,970)	34,770^c^ (17,743–46,913)	71,855^b^ (19,782–133,294)			<0.01
Indole-3-propionic acid	14,629^a, a^ (8,342–41,807)	6,359^b, a^ (273–11,067)	21,609^a, a^ (11,397–213,321)	9,929^a, b^ (6,433–39,621)	4,103^b, b^ (122–8,132)	9,006^a, b^ (5,149–261,311)		0.01	<0.01
Kynurenic acid	827^b^ (541–1,176)	6,188^a^ (388–18,895)	596^b^ (318–1,618)	690^b^ (397–8,980)	8,283^a^ (3,900–34,883)	804^b^ (472–1,830)			<0.01
Tryptophan	30,456^b^ (15,983–142,360)	99,160^a^ (21,844–170,273)	68,305^a^ (32,808–138,909)	40,964^b^ (16,338–83,084)	87,474^a^ (51,120–133,855)	95,811^a^ (31,515–284,375)			<0.01
**Sphingolipid metabolites**									
Cellobiose	15,190^a^ (5,457–29,788)	5,109^b^ (1,738–43,182)	5,063^b^ (2,557–12,603)	13,562^a^ (2,201–25,493)	4,212^b^ (2,808–89,792)	10,658^b^ (1,087–20,781)			0.01
D-erythro-sphingosine	6,805^a^ (2,526–29,549)	3,756^bc^ (975–18,571)	2,036^cd^ (915–5,138)	4,890^ab^ (955–11,023)	1,622^d^ (672–9,162)	4,518^ab^ (3,092–14,801)	0.01		<0.01
Isopentadecanoic acid	53,407^a^ (28,784–91,274)	24,171^c^ (15,867–34,465)	29,321^b^ (4,722–51,680)	51,526^a^ (25,488–95,283)	16,855^c^ (6,681–316,781)	30,903^b^ (16,922–70,276)			<0.01
Pentadecanoic acid	28,576^a^ (14,614–40,686)	14,448^b^ (8,305–31,683)	19,097^b^ (12,240–23,067)	25,694^a^ (17,888–41,885)	14,819^b^ (9,078–27,971)	17,464^b^ (3,677–32,186)			<0.01
Phytosphingosine	6,552^a^ (4,474–14,350)	3,373^bc^ (1,053–36,357)	5,011^bc^ (2,568–5,546)	4,056^c^ (1,714–8,857)	2,686^d^ (1,639–6,759)	5,229^ab^ (3,312–11,865)	0.02		<0.01
**Antioxidants/antimicrobials**									
3-hydroxybenzoic acid	785^a^ (350–2,503)	190^b^ (116–262)	548^a^ (248–2,433)	734^a^ (352–3,362)	171^b^ (113–317)	611^a^ (290–8,417)			<0.01
Hexadecylglycerol NIST	6,021^a, a^ (4,332–11,689)	2,783^c, a^ (620–3,860)	4,273^b, a^ (1,897–5,447)	3,592^a, b^ (2,727–12,350)	1,632^c, b^ (678–3,580)	2,959^b, b^ (1,246–5,882)		0.02	<0.01
3,4-dihydroxycinnamic acid	907^b^ (512–1,275)	1,122^a^ (496–2,884)	689^b^ (434–1,308)	850^b^ (539–1,175)	1,197^a^ (460–1,580)	779^b^ (499–1,069)			<0.01
4-hydroxycinnamic acid	1,636^b^ (1,255–2,227)	8,035^a^ (4,603–23,377)	1,419^b^ (1,027–2,517)	1,465^b^ (1,165–2,443)	13,352^a^ (5,072–33,944)	1,467^b^ (625–2,431)			<0.01
Ferulic acid	1,192^bc^ (652–1,736)	1,920^ab^ (408–4,344)	563^d^ (137–1,194)	861^cd^ (405–1,233)	2,122^a^ (1,300–3,853)	964^bc^ (522–2,479)	0.03		<0.01
Sinapinic acid	793^b, b^ (195–1,046)	1,872^a, b^ (358–4,872)	593^b, b^ (459–776)	776^b, a^ (561–1,769)	2,750^a, a^ (1,008–5,776)	782^b, a^ (531–1,458)		0.02	<0.01
Vanillic acid	733^b^ (326–1,558)	1,979^a^ (451–3,611)	1,359^a^ (605–7,199)	859^b^ (504–11,076)	2,460^a^ (1,577–3,727)	1,725^a^ (458–7,575)			<0.01
**Bacterial energy substrates**									
Ethanolamine	42,429^b^ (264–74,837)	71,841^a^ (570–165,616)	564^c^ (261–46,814)	58,925^b^ (401–95,497)	104,054^a^ (351–229,082)	32,882^c^ (324–57,779)			<0.01
Fucose	179,811^a^ (41,782–233,563)	137,082^a^ (3,849–837,808)	13,332^c^ (8,185–320,277)	137,859^ab^ (50,951–233,808)	68,870^bc^ (1,751–169,428)	120,553^ab^ (17,516–264,172)	0.01		

+ *Feces from one dog (placebo group) unavailable at the days 26–28 time point. P-values were adjusted based on the Benjamini and Hochberg False discovery rate (fdr). Profiles that do not share a common superscript letter differed significantly (fdr-adjusted P < 0.05) based on post-hoc analysis*.

**Figure 5:**
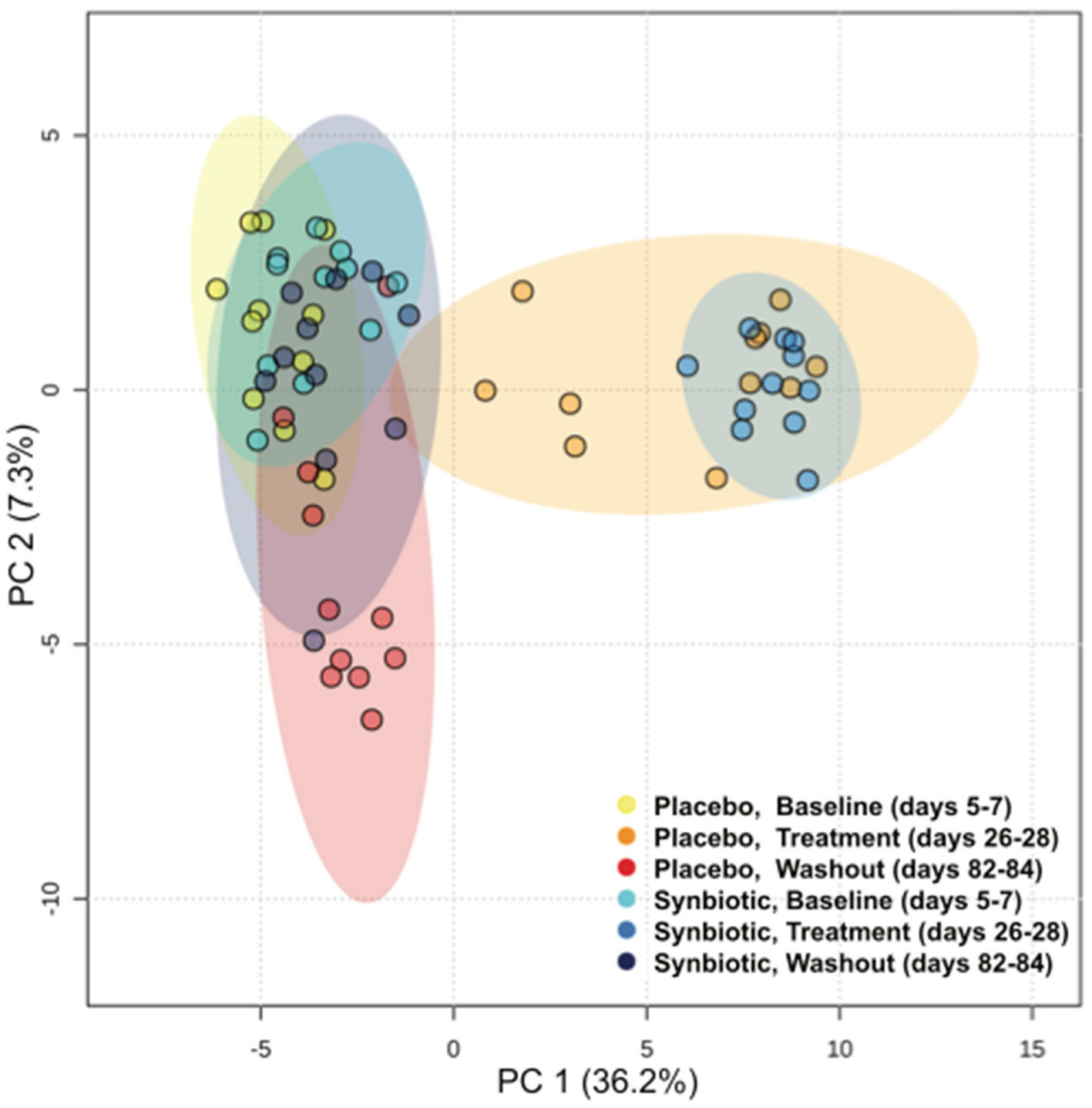
Principal Component Analysis (PCA) of fecal metabolites for dogs that received enrofloxacin/metronidazole followed by placebo or synbiotic for 21 days.

Based on the PCA plot (Figure 5), samples for both treatment groups clustered together at baseline with separate, but overlapping, clustering on days 26–28. Samples from days 82–84 from dogs in the synbiotic group clustered closely with baseline samples for both treatment groups, whereas samples from dogs in the placebo group had little overlap with baseline samples. Visually, metabolites associated with loading axis 1 had changes related to antibiotic administration. These included 4-hydroxycinnaminic acid, short chain fatty acids [including 3-(3-hydroxyphenyl)propionic, 2-aminobutyric, and phenylacetic acid], tryptophan metabolites (indole-3-acetate and kynurenic acid), and bile acids (lithocholic, deoxycholic, and cholic acid). Metabolites aligned with loading axis 2 included cellobiose, energy substrates (particularly fucose and ethanolamine), and additional short-chain fatty acids.

## Discussion

Oral administration of enrofloxacin with metronidazole causes hyporexia, vomiting, and diarrhea in 41, 77, and 100% of healthy dogs, with partial mitigation of signs in dogs also administered synbiotics (5). Based on results of this study, derangements in the fecal microbiome and metabolome secondary to combination enrofloxacin/metronidazole therapy also are severe. Recovery of the fecal microbiome and metabolome differed significantly between treatment groups after antibiotic discontinuation. Significant group-by-time interactions were noted for numerous ASVs associated with eubiosis; SCFA, bile acid, tryptophan, and sphingolipid metabolites; profiles for antioxidants and antimicrobials; and bacterial energy substrate profiles.

Derangements in the fecal microbiome secondary to antibiotic therapy in dogs and cats previously have been reported for a variety of antibiotics (3, 4, 6, 7, 11, 12). Alpha diversity metrics, beta diversity, and the dysbiosis index were significantly altered by clindamycin administration to cats, with recovery occurring between 44 and 603 days after antibiotic discontinuation (4, 6). Alpha diversity metrics, beta diversity, and the dysbiosis index also were significantly altered in healthy dogs by administration of tylosin for 7 days (12). Alpha diversity metrics and the dysbiosis index returned to baseline values 56 days after antibiotic discontinuation, whereas unweighted beta diversity remained significantly altered. Although the dysbiosis index and total observed species did not differ significantly between baseline and the conclusion of washout in that study, derangements in abundance of *C. hiranonis, Faecalibacterium*, and *Turicibacter* persisted. Interestingly, significant effects on the relative abundance of individual ASVs were uncommon—potentially due to use of categorical vs. quantitative statistical analysis.

The impact of metronidazole monotherapy (12.5–15 mg/kg mg/kg BID × 14 days) on the fecal microbiome and metabolome also has been described (7, 11). Alpha and beta diversity were significantly deranged during antibiotic administration in both studies, with complete recovery of alpha diversity 14–28 days after antibiotic discontinuation. Beta diversity, however, remained significantly different from baseline 28 days after antibiotic discontinuation in one of the two studies (7). Abundances of *Bifidobacterium* and G*ammaproteobacteria* increased during antibiotics then returned to baseline levels during washout in both studies, whereas *Bacteroides* decreased during or after antibiotic therapy with variable recovery during washout. The composition of the phylum *Firmicutes* changed significantly during antibiotic administration in both studies. The abundance of *Lactobacillales* significantly increased during antibiotic administration, whereas the abundance of beneficial *Clostridial* species declined during antibiotic administration with recovery thereafter (7, 11). In addition to microbiome derangements, diarrhea was noted in one of the two studies (7).

Alpha and beta diversity, as well as the dysbiosis index, were significantly altered during antibiotic administration for both treatment groups in this study. Consistent with prior reports, total bacteria decreased during antibiotic administration with recovery to baseline abundance during washout. However, recovery of alpha diversity metrics was incomplete at the conclusion of the washout period for dogs in both treatment groups. Beta diversity and the dysbiosis index also remained significantly different from baseline for dogs in the placebo group. Derangements were present in this study for a greater number of genera and other ASVs compared to previous reports from dogs and cats (3, 4, 6, 11, 12), primarily in the same bacterial families and clusters. The most likely explanation for the greater number of ASVs affected is the use of a broader spectrum antibiotic combination. The relative abundances of *Bifidobacterium, Lactobacillus, Streptococcus*, and *Lachnospiraceae* increased during antibiotic administration, whereas decreases were identified for the relative abundances of *Adlercreutzia, Collinsella, Bacteroides, Prevotella*, and members of the *Clostridium* clusters IV and XIV. Persistent or delayed derangements in relative abundance of 16 genera were identified at the conclusion of washout for dogs in the placebo group. Quantitative PCR demonstrated persistent decreases in abundances of *C. hiranonis, Faecalibacterium*, and *Turicibacter*.

Based on results of prior studies, relative abundances for some ASVs might return to baseline abundance in the weeks following antibiotic discontinuation only to decline or overshoot past baseline values over a longer time frame (4, 6, 12). When considering genera with abundances that differed significantly in two studies of clindamycin administration in cats, recovery to baseline values at the terminal sampling time point (44 or 603 days after antibiotic discontinuation, respectively) was concordant for only four of 18 genera. Genera with persistent or delayed derangements in abundance included *Bifidobacterium, Adlercreutzia, Bacteroides, Oscillospira, Ruminococcus, Megasphaera*, and numerous members of the *Clostridium* clusters IV and XIV. Because this study included only one post-antibiotic sampling time point, it was not possible to determine whether ASVs might undergo similar longer-term alterations in dogs. However, for genera with discordant patterns of recovery among reports, results of this study generally matched those of the shorter-term cat study.

Changes in the fecal microbiome in this study also are consistent with results seen in dogs with naturally-occurring acute and chronic enteropathies (9, 13–16). Similarities in results among studies could reflect relatively recent exposure to antibiotics in studies of naturally-occurring enteropathies or a shared underlying pathologic response. It also is possible that adult-onset enteropathies are the clinical manifestation of dysbiosis induced by historical antibiotic exposure alone or in combination with historical gastrointestinal insult (17). Differentiation among these possibilities will require collection of more robust antibiotic histories with inclusion of exposure times and types as covariates in large-scale analyses. Pending those data, it is prudent to avoid unnecessary antibiotic usage and mitigate antibiotic-induced dysbiosis when possible. Synbiotic administration impacted the recovery of the fecal microbiome after antibiotic administration in this study. Beta diversity and the dysbiosis index returned to baseline levels only for dogs administered the synbiotic combination as did abundances for *C. hiranonis, Faecalibacterium*, and *Turicibacter*. Finally, significant group-by-time interactions were noted for 28 ASVs in this study. These changes occurred in spite of discontinuation of the synbiotic combination at the same time as antibiotics, instead of 4–6 weeks thereafter as is often recommended in people.

Antibiotics have been found to markedly derange the fecal metabolome of dogs (7), but the ameliorative effects of synbiotics on antibiotic-induced derangements previously have not been described. Profiles for 86% of identified metabolites were significantly altered by antibiotic therapy, alone or in combination with synbiotics. Although the majority of significant associations in this study were temporal, significant group-by-time interactions or concurrent group and time effects were detected for 24% of metabolites. Furthermore, global fecal metabolite composition significantly differed between treatment groups both during and after antibiotic administration (Figure 5).

Short-chain fatty acids are produced in the large intestine as a result of bacterial carbohydrate fermentation and serve as the preferred energy substrate for colonocytes. However, SCFA also have important roles in increasing T regulatory cell function, modulating the innate immune system, and increasing intestinal barrier function (18). Significant temporal effects, alone or in combination with group-by-time interactions, were identified for numerous SCFA metabolites. This is not particularly surprising given derangements in abundance of *Bifidobacterium, Bacteroides, Prevotella, Lactobacillus, Turicibacter, Megamonas*, and members of the *Clostridium* clusters IV and XIVa (*Blautia, C. hiranonis, Ruminococcus, Peptostreptococcus*, and *Faecalibacterium*)—all of which contribute to SCFA production (19). Derangements were persistent at the conclusion of washout for many ASVs and metabolite profiles. Changes were similar to those described in dogs with acute enteropathy both prior to and after metronidazole therapy (16), as well as up to 603 days after clindamycin administration in cats (4).

Bile acid dysmetabolism also was identified during antibiotic administration in this study. Profiles for the secondary bile acids deoxycholic and lithocholic acid significantly decreased during antibiotics, whereas cholic acid profiles increased. Dysmetabolism is believe to reflect a combination of downregulation of apical sodium-dependent bile acid transporters and decreased deconjugation of primary bile acids due to reduced abundances of *Eubacterium* and *Clostridium* cluster IV and XIVa species, particularly *C. hiranonis* in dogs (7, 14, 15). Ramifications of bile acid dysmetabolism can include diarrhea due to osmotic effects, increased intestinal permeability, and altered immune regulation (14, 15, 20). Bile acid dysmetabolism previously has been described in dogs with both chronic and acute enteropathy, as well as dogs and cats administered antibiotics (4, 6, 7, 14–16). Although changes during antibiotic therapy did not differ between treatment groups in this study, deoxycholic acid profiles normalized after antibiotic discontinuation for the synbiotic group alone with a similar (but non-significant) pattern for lithocholic acid profiles. These results are consistent with prior data showing partial normalization of bile acid profiles in dogs with acute diarrhea treated with fecal transplantation, but not metronidazole, in spite of resolution of clinical signs in both groups (16). In that study, the difference between groups was due to incomplete recovery of *C. hiranonis* in some dogs in the metronidazole treatment group.

Finally, we identified significant antibiotic-induced derangements in tryptophan, sphingolipid, benzoic acid, and cinnaminic acid metabolism. Tryptophan dysmetabolism was characterized by decreased indole profiles in association with increased tryptophan and kynurenic acid profiles. Both indole products and kynurenic acid increase immune function and epithelial restitution, albeit *via* different mechanisms (21). Indole products also increase pathogen resistance *via* modulation of bacterial virulence factors (21). Although indole-3-acetate and indole-3-proprionic acid profiles returned to baseline after antibiotic discontinuation, recovery was incomplete or absent for indole-3-lactate and tryptophan, suggesting ongoing dysmetabolism. Sphingolipids are important components of the apical cell membrane of intestinal epithelial cells, differentially distributed between villus and crypt cells (22). Dysregulation of sphingolipid production is associated with increased intestinal permeability and a shift from an anti-inflammatory to pro-inflammatory state in mouse models of chronic enteropathy, as well as individuals with Crohn's disease and inflammatory bowel disease (22). Derangements in several sphingolipid metabolites persisted after antibiotic discontinuation for both treatment groups in this study. The exception was D-erythro-sphingosine profiles, which normalized after antibiotic discontinuation for dogs in the synbiotic group. Finally, antibiotic administration significantly affected benzoic and cinnaminic acid profiles, both of which have antimicrobial effects (23, 24). Cinnaminic acids also affect management of obesity and diabetes mellitus (24). Recovery after antibiotic discontinuation was mixed for cinnaminic acid metabolites. Persistent tryptophan, sphingolipid, and cinnaminic acid dysmetabolism have been identified over 600 days after clindamycin administration to cats (4), suggesting potentially irreversible effects of antibiotics on metabolic pathways.

One additional finding of this study was significant alterations in ethanolamine and fucose profiles during antibiotic therapy. A key facet of colonization resistance is the ability of commensal bacteria to efficiently process nutrients, such as fucose, to outcompete pathogenic species (25). Inflammation, however, increases exposure of phosphatidylethanolamine from intestinal cell membranes, which is converted to ethanolamine in the lumen (26). Although commensal bacteria have poor ability to metabolize ethanolamine, pathogenic bacteria can alter their metabolism to increase use of ethanolamine, allowing them to rapidly expand and colonize the gut (26, 27). The trigger for this metabolic shift in adherent-invasive *E. coli* is increased propanediol concentrations (28), such as were found in this study. Synbiotic administration was associated with normalization of fucose profiles, whereas fucose profiles decreased after antibiotic discontinuation for the placebo group. Further study is required to determine whether the latter is partially responsible for the incomplete recovery of commensal bacteria, such as *C. hiranonis*, after antibiotic exposure for dogs not administered synbiotics.

The use of healthy research dogs with a uniform diet, husbandry, and environment could be considered a limitation of this study. However, findings were remarkably similar to prior reports of privately-owned healthy dogs administered antibiotics as well as dogs with naturally-occurring gastrointestinal disease (12, 16). Although longer than that used in most prior studies (11, 12, 29), follow-up after antibiotic discontinuation was limited and included only one time point. Given previously identified patterns of overshoot or decline after initial normalization of taxa and metabolites (4, 6, 12), future studies with longer-term follow-up will be necessary to determine the full ramifications of historical antibiotic exposure. Long-term cohort studies of privately-owned animals likely will be required to elucidate the clinical ramifications of persistent antibiotic-induced derangements in the microbiome and/or metabolome. Other limitations of this study include lack of targeted metabolite analyses and lack of characterization of archaea, fungi, protists, and viruses, all of which contribute to host-microbiome interactions (30, 31).

## Conclusions

Broad-spectrum antibiotic regimens in dogs are associated with a high incidence of AAGS. Adverse clinical effects are believed to be due to negative effects of antibiotics on the gastrointestinal microbiome, leading to alterations in the metabolome and opportunistic colonization by pathogenic bacteria. Based on results of this study, derangements in the fecal microbiome and metabolome secondary to combination enrofloxacin/metronidazole therapy are profound. Recovery of the fecal microbiome and metabolome composition overall after antibiotic discontinuation was greater for dogs administered synbiotics. Significant group-by-time interactions also were noted for numerous ASVs associated with eubiosis; SCFA, bile acid, tryptophan, and sphingolipid metabolites; antioxidants and antimicrobials; and bacterial energy substrates. Further study is warranted to determine the long-term clinical ramifications of differences in antibiotic-induced dysbiosis and dysmetabolism between dogs administered antibiotics alone vs. in combination with synbiotics. Pending those studies, administration of a synbiotic combination 1–2 h after each antibiotic dosage is warranted to minimize AAGS as well as derangements in the fecal microbiome and metabolome.

## Data Availability Statement

The datasets presented in this study can be found in online repositories. The names of the repository/repositories and accession number(s) can be found at: https://www.ncbi.nlm.nih.gov/, SRP165850 and https://www.metabolomicsworkbench.org/, 10.21228/M8RH7J, Project ID: PR001013.

## Ethics Statement

The animal study was reviewed and approved by Institutional Animal Care and Use Committee of the University of Tennessee, Knoxville (protocol 2544).

## Author Contributions

JW: Developed the hypothesis and study design. JW and TM: Organized and conducted the experiment. JW, JP, and JS: Interpreted and analyzed the results. JW, JP, TM, and JS: Wrote and revised the manuscript. All authors contributed to the article and approved the submitted version.

## Conflict of Interest

JW and JS have received past honoraria from Nutramax Laboratories Veterinary Sciences, Inc. for development of educational materials and public speaking. The remaining authors declare that the research was conducted in the absence of any commercial or financial relationships that could be construed as a potential conflict of interest.
