# Supplementary material for: Effects of Synbiotics on the Fecal Microbiome and Metabolomic Profiles of Healthy Research Dogs Administered Antibiotics: A Randomized, Controlled Trial
Source: Front Vet Sci. 2021 May 26;8:665713. doi: 10.3389/fvets.2021.665713 (PMC8187564; doi:10.3389/fvets.2021.665713)
Supplement: Supplementary file 1 [file Table_1.docx]

**Supplementary Table 1.** Median percent abundance (range) for bacterial taxa that significantly differed for dogs that received enrofloxacin/metronidazole followed by placebo or synbiotic for 21 days. Relative abundances that do not share a common superscript letter differed significantly (fdr-adjusted *P* < 0.05) based on post-hoc analysis.

|  | **Placebo** | | | **Synbiotic** | | | **fdr *P*-value** | | |
| --- | --- | --- | --- | --- | --- | --- | --- | --- | --- |
|  | Baseline | Days 26-28 | Days 82-84 | Baseline | Days 26-28 | Days 82-84 | Group* Time | Group | Time |
| ***Actinobacteria*** | 1.95^c^  (0.59-4.79) | 8.22^a^  (2.35-19.04) | 6.81^b^  (1.35-11.46) | 2.71^c^  (0.47-6.75) | 17.23^a^  (2.76-20.19) | 4.53^b^  (0.69-13.98) |  |  | <0.01 |
| **- Actinobacteria** | 0.12^b^  (0-2.73) | 12.6^a^  (3.21-19.04) | 0.81^b^  (0-5.46) | 0.13^b^  (0-2.74) | 9.32^a^  (0-17.8) | 0.69^b^  (0-4.75) |  |  | <0.01 |
| ** Bifidobacteriales* | 0.51^c^  (0-2.74) | 8.22^a^  (0-19.04) | 1.57^b^  (0-5.46) | 0^c^  (0-1.72) | 15.65^a^  (2.61-17.8) | 0.58^b^  (0-4.75) | 0.01 |  | <0.01 |
| - Bifidobacteriaceae | 0.51^bc^  (0-2.74) | 8.22^a^  (0-19.04) | 1.57^b^  (0-5.46) | 0^d^  (0-1.72) | 15.65^a^  (2.61-17.8) | 0.58^cd^  (0-4.75) | 0.01 |  | <0.01 |
| * Bifidobacterium | 0.51^bc^  (0-2.74) | 8.22^a^  (0-19.04) | 1.57^b^  (0-5.46) | 0^d^  (0-1.72) | 15.65^a^  (2.61-17.8) | 0.58^cd^  (0-4.75) | 0.01 |  | <0.01 |
| **- Coriobacteriia** | 1.66^b^  (0.47-6.57) | 0.51^c^  (0-2.9) | 4.53^a^  (1.31-8.5) | 1.51^b^  (0.47-6.75) | 0.96^c^  (0-2.38) | 2.71^a^  (0.69-9.23) |  |  | <0.01 |
| ** Coriobacteriales* | 1.37^b^  (0.47-3.16) | 0.51^c^  (0-2.38) | 2.97^a^  (1.31-7.66) | 2.29^b^  (0.47-6.75) | 1.34^c^  (0-2.9) | 4.39^a^  (0.69-9.23) |  |  | <0.01 |
| - Coriobacteriaceae | 1.37^b^  (0.47-3.16) | 0.51^c^  (0-2.38) | 2.97^a^  (1.31-7.66) | 2.29^b^  (0.47-6.75) | 1.34^c^  (0-2.9) | 4.39^a^  (0.69-9.23) |  |  | <0.01 |
| * g___ | 0^b^  (0-0.82) | 0^b^  (0-0) | 1.31^a^  (0-5.57) | 0^b^  (0-0) | 0^b^  (0-0) | 0^b^  (0-8.58) | <0.01 | <0.01 | <0.01 |
| * Adlercreutzia | 0.19^ab^  (0-0.55) | 0^c^  (0-0) | 0^b^  (0-0.73) | 0^bc^  (0-0.55) | 0^c^  (0-0) | 0.36^a^  (0-1.29) | 0.03 |  | <0.01 |
| * Collinsella | 1.06^a,β^  (0.4-2.62) | 0.51^b,β^  (0-2.38) | 0.58^a,β^  (0-6.86) | 1.79^a,α^  (0.47-6.75) | 1.34^b,α^  (0-2.9) | 3.25^a,α^  (0.33-6.81) |  | 0.01 | 0.04 |
| * Slackia | 0^bc^  (0-0.13) | 0^c^  (0-0) | 0^c^  (0-0) | 0^b^  (0-0.3) | 0^c^  (0-0) | 0.24^a^  (0-0.4) | 0.01 | 0.03 | 0.01 |
|  |  |  |  |  |  |  |  |  |  |
| ***Bacteroidetes*** | 18.63^a,α^  (6.36-30.48) | 0.22^c,α^  (0-10.9) | 20.18^b,α^  (0.97-31.88) | 19.11^a,β^  (0.43-29.42) | 0^c,β^  (0-0.18) | 3.41^b,β^  (0-9.27) |  | 0.01 | <0.01 |
| **- Bacteroidia** | 17.67^a^  (0.43-30.48) | 0^c^  (0-7.53) | 6.09^b^  (0-31.88) | 20.07^a^  (10-29.42) | 0.1^c^  (0-10.9) | 5.6^b^  (0.97-27.72) |  |  | <0.01 |
| ** Bacteroidales* | 18.63^a,α^  (6.36-30.48) | 0.22^c,α^  (0-10.9) | 20.18^b,α^  (0.97-31.88) | 19.11^a,β^  (0.43-29.42) | 0^c,β^  (0-0.18) | 3.41^b,β^ (0-9.27) |  | <0.01 | <0.01 |
| - Bacteroidaceae | 5.11^a^  (0.61-12.81) | 0^c^  (0-6.54) | 6.8^b^  (0-13.1) | 6.12^a^  (0-17.99) | 0^c^  (0-0.18) | 1.65^b^  (0-6.67) |  |  | <0.01 |
| * Bacteroides | 5.11^a^  (0.61-12.81) | 0^c^  (0-6.54) | 6.8^b^  (0-13.1) | 6.12^a^  (0-17.99) | 0^c^  (0-0.18) | 1.65^b^  (0-6.67) |  |  | <0.01 |
| - Prevotellaceae | 3.97 ^a,α^  (0.59-6.89) | 0 ^c,α^  (0-3.14) | 1.08 ^b,α^  (0.22-4.96) | 2.6^a,β^  (0-9.71) | 0^c,β^  (0-0) | 0^b,β^  (0-2.16) |  | 0.01 | <0.01 |
| * Prevotella | 0.27 ^a,α^  (0-1.34) | 0 ^c,α^  (0-0) | 0 ^b,α^  (0-3.39) | 0.22^a,β^  (0-0.64) | 0^c,β^  (0-0.1) | 0^b,β^  (0-1.21) |  |  | <0.01 |
| - S24_7 | 6.73^ab^  (1.97-13.7) | 0^d^  (0-4.4) | 1.67^a^  (0-7.27) | 2.67^bc^  (0.12-12.22) | 0^d^  (0-0) | 0^cd^  (0-2.94) | 0.01 | <0.01 | <0.01 |
| * g___ | 1.5^ab^  (0-9.73) | 0^d^  (0-0.67) | 8.39^a^  (0-15.2) | 0.16^bc^  (0-8.82) | 0^d^  (0-0) | 0^cd^  (0-7.05) | 0.02 | <0.01 | <0.01 |
| - [Paraprevotellaceae] | 7.39^a,α^  (2.18-14.07) | 0^c,α^  (0-4.4) | 1.67^b,α^  (0.47-8.54) | 2.93^a,β^  (0.12-12.44) | 0^c,β^  (0-0.1) | 0.72^b,β^  (0-3.24) |  | <0.01 | <0.01 |
| * g___ | 0.27^a^  (0-1.34) | 0^b^  (0-0) | 0^a^  (0-3.39) | 0.22^a^  (0-0.64) | 0^b^  (0-0.1) | 0^a^  (0-1.21) |  |  | <0.01 |
| * [Prevotella] | 6.73^a,α^  (1.97-13.7) | 0^c,α^  (0-4.4) | 1.67^b,α^  (0-7.27) | 2.67^a,β^  (0.12-12.22) | 0^c,β^  (0-0) | 0^b,β^  (0-2.94) |  | <0.01 | <0.01 |
|  |  |  |  |  |  |  |  |  |  |
| ***Firmicutes*** | 64.62^b^  (46.15-81.31) | 88.53^a^  (71.55-94.76) | 66.8^b^  (49.2-87.16) | 57.75^b^  (44.63-92.6) | 82.77^a^  (79.81-96.84) | 79.11^a^  (70.93-94.58) | 0.04 |  | <0.01 |
| **- Bacilli** | 9.31^b^  (0-22.52) | 53.09^a^  (37.76-71.04) | 5.68^b^  (1.07-26.9) | 7.5^b^  (2.47-27.1) | 54.73^a^  (3.63-85.62) | 9.05^b^  (0.4-36.14) |  |  | <0.01 |
| ** Lactobacillales* | 0.98^b^  (0.15-12.63) | 59.16^a^  (3.01-85.62) | 1.12^b^  (0.27-7.96) | 2.61^b^  (0-23.85) | 52.43^a^  (37.76-73.81) | 7.56^b^  (0-24.1) |  |  | <0.01 |
| - Enterococcaceae | 0^b^  (0-0) | 0^a^  (0-5.17) | 0^b^  (0-0.68) | 0^b^  (0-0) | 1.71^a^  (0-6.91) | 0^b^  (0-0.35) |  |  | <0.01 |
| * Enterococcus | 0^b^  (0-0) | 0^a^  (0-5.17) | 0^b^  (0-0) | 0^b^  (0-0) | 1.71^a^  (0-3.75) | 0^b^  (0-0) |  |  | <0.01 |
| - Lactobacillaceae | 0.48^b^  (0-11.24) | 32.7^a^  (0-54.52) | 0.4^b^  (0-1.65) | 0.5^b^  (0-23.85) | 25.96^a^  (18.31-50.35) | 0.41^b^  (0-7.56) |  |  | <0.01 |
| * Lactobacillus | 0.48^b^  (0-11.24) | 32.7^a^  (0-54.52) | 0.4^b^  (0-1.65) | 0.5^b^  (0-23.85) | 25.96^a^  (18.31-50.35) | 0.41^b^  (0-7.56) |  |  | <0.01 |
| - Streptococcaceae | 0.19^c^  (0-1.48) | 26.89^a^  (0-41.4) | 0.4^b^  (0-6.88) | 0^c^  (0-4.83) | 18.42^a^  (3.59-36.64) | 5.31^b^  (0-17.5) |  |  | <0.01 |
| * Streptococcus | 0.19^c^  (0-1.48) | 26.89^a^  (0-41.4) | 0.4^b^  (0-6.88) | 0^c^  (0-4.83) | 18.42^a^  (3.59-36.64) | 5.31^b^  (0-17.5) |  |  | <0.01 |
| ** Turicibacterales* | 5.9^ab^  (1.38-13.21) | 0^d^  (0-0.62) | 2.18^c^  (0-8.86) | 3.25^bc^  (0-19.05) | 0^d^  (0-0.27) | 9.43^a^  (1.16-25.61) | <0.01 |  | <0.01 |
| - Turicibacteraceae | 5.9^ab^  (1.38-13.21) | 0^d^  (0-0.62) | 2.18^c^  (0-8.86) | 3.25^bc^  (0-19.05) | 0^d^  (0-0.27) | 9.43^a^  (1.16-25.61) | <0.01 |  | <0.01 |
| * Turicibacter | 5.9^ab^  (1.38-13.21) | 0^d^  (0-0.62) | 2.18^c^  (0-8.86) | 3.25^bc^  (0-19.05) | 0^d^  (0-0.27) | 9.43^a^  (1.16-25.61) | <0.01 |  | <0.01 |
| **- Clostridia** | 36.82^a^  (13.23-58.55) | 18.07^b^  (6.63-29.49) | 28.4^a^  (8.7-58.75) | 33.57^a^  (10.83-42.92) | 20.16^b^  (2.39-47.74) | 35.75^a^  (12.23-58.79) |  |  | <0.01 |
| ** Clostridiales* | 29.8^bc^  (13.23-42.68) | 20.16^d^  (2.39-47.74) | 22.59^cd^  (8.7-58.79) | 41.44^ab^  (10.83-58.55) | 19.01^d^  (6.63-26.09) | 42.57^a^  (28.01-58.75) | 0.01 | 0.04 | <0.01 |
| - ___ | 0.45^b^  (0-2.66) | 0^c^  (0-0.01) | 0.48^a^  (0-4.8) | 0^b^  (0-0.87) | 0^c^  (0-0) | 1.11^a^  (0-2.28) |  |  | <0.01 |
| - Clostridiaceae | 7.71^b^  (6.23-15.02) | 0.56^d^  (0-5.08) | 4.23^c^  (0.91-22.37) | 11.74^ab^  (2.6-21.61) | 0.24^d^  (0-1.07) | 14.14^a^  (7.59-26.59) | <0.01 |  | <0.01 |
| * ___ | 7.4^ab^  (5.66-14.95) | 0.4^c^  (0-0.98) | 2.73^b^  (0.91-17.52) | 9.68^a^  (0.58-18.58) | 0^c^  (0-0.4) | 11.99^a^  (5.97-16.48) | 0.01 |  | <0.01 |
| * g___ | 0.29^a^  (0-1.91) | 0^b^  (0-5.08) | 0.64^a^  (0-2.78) | 1.49^a^  (0-3.02) | 0^b^  (0-1.07) | 1.63^a^  (0-3.04) |  |  | 0.01 |
| * Clostridium | 0^c^  (0-0.14) | 0^c^  (0-0.7) | 0^bc^  (0-2.08) | 0^ab^  (0-3.61) | 0^c^  (0-0) | 0.57^a^  (0-10.36) | 0.05 |  | <0.01 |
| - Lachnospiraceae | 10.49^β^  (4.38-19.51) | 18.56^β^  (2.1-41.37) | 10.1^β^  (0.06-32.31) | 17.5^α^  (3.63-34.92) | 18.52^α^  (6.63-25.75) | 21.3^α^  (10.7-28.87) |  | 0.01 |  |
| * ___ | 3.78^bc^  (1-5.6) | 5.65^a^  (2.1-13.67) | 1.68^c^  (0-9.23) | 3.2^bc^  (1.29-8.72) | 4.57^ab^  (1.07-16.09) | 5.88^a^  (3.42-9.01) | 0.01 |  |  |
| * Blautia | 3.47^β^  (0.54-8.09) | 2.93^β^  (0-16.39) | 3.12^β^  (0-15.33) | 9.36^α^  (0-18.48) | 3.98^α^  (0.5-11.03) | 10.31^α^  (2.03-15.09) |  | 0.03 |  |
| * [Ruminococcus] | 2.68^b^  (0-4.8) | 3.5^ab^  (0-14.16) | 5.07^a^  (0-13.45) | 5.08^a^  (2.16-8.94) | 2.47^ab^  (0-19.82) | 4.67^a^  (2.26-8.26) | 0.05 |  |  |
| - Peptococcaceae | 0.53^a^  (0-1.28) | 0^b^  (0-0.11) | 0^b^  (0-1.39) | 0.38^a^  (0-2.33) | 0^b^  (0-0) | 0.92^a^  (0-2.38) | 0.01 |  | <0.01 |
| * Peptococcus | 0.53^a^  (0-1.28) | 0^b^  (0-0.11) | 0^b^  (0-1.39) | 0.38^a^  (0-2.33) | 0^b^  (0-0) | 0.92^a^  (0-2.38) | 0.01 |  | <0.01 |
| - Peptostreptococcaceae | 0^b^  (0-3.64) | 0^b^  (0-0.3) | 1.19^a^  (0-6.82) | 0^b^  (0-2.2) | 0^b^  (0-0) | 0^a^  (0-8.98) |  |  | <0.01 |
| * Peptostreptococcus | 0^b,α^  (0-0.28) | 0^b,α^  (0-0.3) | 0.96^a,α^  (0-6.82) | 0^b,β^  (0-0) | 0^b,β^  (0-0) | 0^a,β^  (0-7.59) |  | 0.02 | <0.01 |
| - Ruminococcaceae | 4.85^a^  (1.38-10.22) | 0^c^  (0-1.32) | 0.93^b^  (0.51-2.59) | 2.8^a^  (0.77-11.15) | 0^c^  (0-0.11) | 1.16^b^  (0.02-7.12) |  |  | <0.01 |
| * g___ | 1.34^a,α^  (0-2.74) | 0 ^b,α^  (0-0.87) | 0.57^a,α^  (0.22-1.27) | 0.23^a,β^  (0-1.83) | 0^b,β^  (0-0) | 0.27^a,β^  (0-2.02) |  | 0.03 | <0.01 |
| * Faecalibacterium | 3.12^a^  (0.53-8.38) | 0^c^  (0-1.32) | 0^b^  (0-2.18) | 2.44^a^  (0-10.02) | 0^c^  (0-0.11) | 0.94^b^  (0-5.1) |  |  | <0.01 |
| - Veillonellaceae | 3^a^  (0.46-7.27) | 0^c^  (0-1.13) | 0.36^b^  (0-1.96) | 1.47^a^  (0.6-7.62) | 0^c^  (0-0.24) | 0.44^b^  (0-2.64) |  |  | <0.01 |
| * Megamonus | 1.05^a^  (0-3.72) | 0^c^  (0-0) | 0^b^  (0-1.96) | 0.76^a^  (0-4.64) | 0^c^  (0-0.24) | 0.37^b^  (0-1.64) |  |  | <0.01 |
| * Phascolarctobacterium | 1.51^a^  (0.4-2.75) | 0^c^  (0-0.59) | 0.07^b^  (0-1.21) | 0.64^a^  (0-2.69) | 0^c^  (0-0) | 0^b^  (0-1.01) |  |  | <0.01 |
| **- Erysipelotrichi** | 21.4^b^  (8.03-52.17) | 11.7^c^  (1.88-23.98) | 29.63^a^  (17.01-49.3) | 15.63^b^  (9.45-31.03) | 9.9^c^  (0.27-21.6) | 27.97^a^  (10.15-40.62) |  |  | <0.01 |
| ** Erysipelotrichales* | 23.77^b^  (8.03-52.17) | 6.99^d^  (1.88-21.92) | 39.42^a^  (27.97-49.3) | 15.69^bc^  (10.84-31.03) | 11.7^cd^  (0.27-23.98) | 17.09^b^  (10.15-40.62) | 0.01 |  | <0.01 |
| - Erysipelotrichaceae | 23.77^b^  (8.03-52.17) | 6.99^d^  (1.88-21.92) | 39.42^a^  (27.97-49.3) | 15.69^bc^  (10.84-31.03) | 11.7^cd^  (0.27-23.98) | 17.09^b^  (10.15-40.62) | 0.01 |  | <0.01 |
| * g___ | 0.48^ab^  (0.11-1.79) | 0^cd^  (0-1.39) | 0.18^bc^  (0-3.06) | 1.02^a^  (0-3.11) | 0^d^  (0-0) | 1.21^a^  (0-4.22) | 0.01 |  | <0.01 |
| * Allobaculum | 19.85^b^  (0.56-51.64) | 5.39^cd^  (1.88-21.72) | 39.42^a^  (4.07-49.3) | 4.2^d^  (0-29.34) | 11.2^bc^  (0-22.04) | 2.09^d^  (1.12-40.59) | <0.01 | 0.02 |  |
| * Catenibacterium | 0.67^a,β^  (0-8.1) | 0^b,β^  (0-4.84) | 0^a,β^  (0-11.62) | 6.17^a,α^  (0-12.65) | 0.23^b,α^  (0-2.32) | 4.48^a,α^  (0-16.53) |  | 0.01 | <0.01 |
| * [Eubacterium] | 0.89^a^  (0.4-5.16) | 0.07^b^  (0-10.31) | 0^b^  (0-9.22) | 4.88^a^  (0.5-6.45) | 0^b^  (0-0.27) | 4.93^a^  (0-10.86) | <0.01 | 0.03 | <0.01 |
|  |  |  |  |  |  |  |  |  |  |
| ***Fusobacteria*** | 12^a^  (6.39-18.91) | 0^c^  (0-14.44) | 5.57^b^  (1.38-11.9) | 12^a^  (0-27.67) | 0^c^  (0-0.3) | 9.26^b^  (0-17.19) |  |  | <0.01 |
| **- Fusobacteriia** | 11.67 ^a,β^  (0-18.91) | 0^c,β^  (0-0.36) | 5.57^b,β^  (0-11.9) | 12.27^a,α^  (10.23-27.67) | 0^c,α^  (0-14.44) | 9.05^b,α^  (0.68-17.19) |  | 0.02 | <0.01 |
| ** Fusobacteriales* | 12^a^  (6.39-18.91) | 0^c^  (0-14.44) | 5.57^b^  (1.38-11.9) | 12^a^  (0-27.67) | 0^c^  (0-0.3) | 9.26^b^  (0-17.19) |  |  | <0.01 |
| - Fusobacteriaceae | 12^a^  (6.39-18.91) | 0^c^  (0-14.44) | 5.57^b^  (1.38-11.9) | 12^a^  (0-27.67) | 0^c^  (0-0.3) | 9.26^b^  (0-17.19) |  |  | <0.01 |
| * Fusobacterium | 12^a^  (6.39-18.91) | 0^c^  (0-14.44) | 5.57^b^  (1.38-11.9) | 11.96^a^  (0-21.48) | 0^c^  (0-0.3) | 9.26^b^  (0-17.19) |  |  | <0.01 |
|  |  |  |  |  |  |  |  |  |  |
| ***Proteobacteria*** | 2.2^a^  (0.63-4.59) | 0.76^b^  (0-4.51) | 1.61^ab^  (0-11.4) | 2.89^a^  (0.4-7.67) | 0.17^b^  (0-6.85) | 1.68^ab^  (0-3.72) |  |  | 0.01 |
| **- Gammaproteobacteria** | 1.53^a^  (0.34-4.06) | 0^c^  (0-0) | 0^b^  (0-2.78) | 0.91^a^  (0.29-6.82) | 0^c^  (0-0) | 0^b^  (0-3.55) |  |  | <0.01 |
| ** Aeromonadales* | 1.41^ab^  (0.34-3.54) | 0^c^  (0-0) | 0^c^  (0-1) | 1.61^a^  (0.29-6.75) | 0^c^  (0-0) | 1.44^b^  (0-3.43) | 0.01 | <0.01 | <0.01 |
| - Succinivibrionaceae | 1.41^ab^  (0.34-3.54) | 0^c^  (0-0) | 0^c^  (0-1) | 1.61^a^  (0.29-6.75) | 0^c^  (0-0) | 1.44^b^  (0-3.43) | 0.01 | <0.01 | <0.01 |
| * g___ | 0.66^a^  (0.25-1.96) | 0^b^  (0-0) | 0^b^  (0-1) | 0.82^a^  (0-4.06) | 0^b^  (0-0) | 0.69^a^  (0-2.78) | 0.04 |  | <0.01 |

**Supplementary Table 2.** Median (range) peak height of metabolites with profiles that had significant time effects for dogs that received enrofloxacin/metronidazole followed by placebo or synbiotic for 21 days. Profiles that do not share a common superscript letter differed significantly (fdr-adjusted *P* < 0.05) based on post-hoc analysis.

|  | **Placebo** | | | **Synbiotic** | | | fdr *P*-value |
| --- | --- | --- | --- | --- | --- | --- | --- |
|  | Baseline | Days 26-28 | Days 82-84 | Baseline | Days 26-28 | Days 82-84 |  |
| 1,2-anhydro-myo-inositol NIST | 8,502^a^  (1,753-12,438) | 2,565^b^  (882-4,274) | 1,799^b^  (923-11,209) | 4,922^a^  (218-11,477) | 2,876^b^  (269-4,098) | 3,237^b^  (162-19,580) | 0.01 |
| 1-monoolein | 53,341^a^  (27,772-151,699) | 13,140^b^  (7,204-61,168) | 61,948^a^  (25,605-94,112) | 37,397^a^  (19,925-113,553) | 17,215^b^  (721-60,356) | 48,582^a^  (21,777-113,840) | <0.01 |
| 1-monopalmitin | 4,427^a^  (370-10,781) | 2,466^b^  (478-6,193) | 4,113^a^  (2,261-7,871) | 4,056^a^  (701-8,578) | 2,111^b^  (1,066-7,028) | 5,760^a^  (1,839-10,482) | <0.01 |
| 2,4-diaminobutyric acid | 5,551^a^  (1,401-9,021) | 1,223^c^  (417-12,349) | 2,661^b^  (1,021-3,656) | 5,124^a^  (1,888-7,023) | 1,042^c^  (523-2,201) | 2,845^b^  (903-5,453) | <0.01 |
| 2,5-dihydroxypyrazine NIST | 1,868^a^  (1,346-2,494) | 1,344^b^  (791-2,975) | 1,401^b^  (1,159-2,107) | 1,910^a^  (1,522-3,263) | 1,509^b^  (740-2,490) | 1,434^b^  (1,127-2,219) | <0.01 |
| 2-aminobutyric acid | 90,013^b^  (60,021-161,873) | 20,331^c^  (3,904-96,172) | 130,033^a^  (44,028-344,232) | 122,729^b^  (36,089-212,264) | 8,978^c^  (5,239-62,097) | 136,646^a^  (85,394-326,247) | <0.01 |
| 2-aminophenol | 862^a^  (472-1,257) | 324^c^  (174-579) | 480^b^  (308-1,029) | 834^a^  (376-1,904) | 449^c^  (189-577) | 518^b^  (230-1,233) | <0.01 |
| 2-deoxyerythritol | 3,301^a^  (1,526-4,499) | 1,741^b^  (790-3,716) | 2,806^a^  (1,106-4,401) | 3,564^a^  (1,741-10,028) | 1,790^b^  (792-4,313) | 3,725^a^  (1,020-5,744) | <0.01 |
| 2-deoxyguanosine | 2,017^a^  ( 727-5,591) | 392^b^  (50-1,511) | 1,956^a^  (600-5,646) | 983^a^  (514-2,580) | 364^b^  (133-666) | 971^a^  (433-9,222) | <0.01 |
| 2-deoxytetronic acid | 4,042^a^  (1,569-22,356) | 1,157^b^  (186-14,752) | 2,324^a^  (1,540-8,108) | 5,048^a^  (1,250-49,806) | 892^b^  (526-1,978) | 3,829^a^  (1,140-14,386) | <0.01 |
| 2-hydroxybutanoic acid | 5,994^b^  (3,221-29,404) | 12,674^b^  (4,825-22,146) | 24,725^a^  (9,275-51,409) | 7,342^b^  (3,971-113,729) | 9,169^b^  (1,147-37,955) | 16,023^a^  (6,796-46,386) | <0.01 |
| 2-hydroxyhexanoic acid | 4,827^c^  (1,055-22,004) | 49,507^a^  (27,380-102,158) | 23,342^b^  (977-48,940) | 6,228^c^  (3,393-40,431) | 62,531^a^  (34,636-124,854) | 14,360^b^  (4,423-59,479) | <0.01 |
| 2-hydroxyvaleric acid | 4,623^a^  (3,406-5,736) | 1,476^b^  (540-11,370) | 4,689^a^  (1,896-6,488) | 4,375^a^  (2,677-21,425) | 965^b^  (597-9,128) | 4,067^a^  (1,883-8,770) | <0.01 |
| 2-picolinic acid | 2,286^a^  (1,751-3,216) | 1,406^b^  (1,052-2,878) | 2,157^a^  (1,197-2,732) | 2,227^a^  (528-4,390) | 1,251^b^  (966-2,254) | 2,230^a^  (758-4,489) | <0.01 |
| 3-(3-hydroxyphenyl)propionic acid | 261,529^a^  (149,818-410,768) | 1,879^c^  (1,325-26,908) | 127,896^b^  (1,944-250,573) | 129,710^a^  (3,303-569,367) | 1,527^c^  (924-4,003) | 142,548^b^  (14,129-321,723) | <0.01 |
| 3,4-dihydroxycinnamic acid | 907^b^  (512-1,275) | 1,122^a^  (496-2,884) | 689^b^  (434-1,308) | 850^b^  (539-1,175) | 1,197^a^  (460-1,580) | 779^b^  (499-1,069) | <0.01 |
| 3-(4-hydroxyphenyl)propionic acid | 89,461^b^  (18,211-355,326) | 26,006^c^  (8,474-99,922) | 269,044^a^  (45,812-687,935) | 121,925^b^  (28,869-268,775) | 20,974^c^  (6,288-35,824) | 155,582^a^  (86,037-894,720) | <0.01 |
| 3,6-anhydro-D-galactose | 5,251^a^  (3,611-7,881) | 1,644^b^  (1,347-3,976) | 1,669^b^  (1,397-4,055) | 5,338^a^  (3,480-7,423) | 1,802^b^  (729-2,555) | 1,655^b^  (1,288-3,073) | <0.01 |
| 3,6-anhydro-D-glucose | 4,504^a^  (3,182-6,778) | 1,766^b^  (1,167-3,509) | 1,591^b^  (1,160-3,657) | 4,632^a^  (3,377-6,704) | 1,453^b^  (1,072-2,045) | 1,595^b^  (442-2,864) | <0.01 |
| 3-aminoisobutyric acid | 7,459^a^  (3,499-36,854) | 5,247^b^  (210-8,123) | 32,758^a^  (845-55,405) | 7,710^a^  (3,770-20,700) | 3,380^b^  (460-12,971) | 2,883^a^  (2,204-78,482) | 0.01 |
| 3-hydroxy-3-methylglutaric acid | 190^b^  (95-371) | 1,214^a^  (377-4,425) | 160^c^  (103-304) | 209^b^  (83-305) | 878^a^  (503-3,704) | 116^c^  (96-240) | <0.01 |
| 3-hydroxybenzoic acid | 785^a^  (350-2,503) | 190^b^  (116-262) | 548^a^  (248-2,433) | 734^a^  (352-3,362) | 171^b^  (113-317) | 611^a^  (290-8,417) | <0.01 |
| 3-hydroxybutyric acid | 7,184^a^  (3,027-10,079) | 2,712^b^  (237-11,586) | 7,454^a^  (2,366-13,042) | 10,391^a^  (3,067-50,349) | 1,618^b^  (443-7,224) | 4,380^a^  (1,754-46,165) | <0.01 |
| 4-aminobutyric acid | 6,834^a^  (5,180-11,170) | 1,610^b^  (718-10,973) | 2,702^b^  (190-5,965) | 5,889^a^  (2,841-20,586) | 3,310^b^  (1,330-10,461) | 3,585^b^  (1,658-6,878) | <0.01 |
| 4-hydroxybutyric acid | 1,814^b^  (1,170-10,221) | 3,859^a^  (2,692-7,318) | 3,519^b^  (1,627-6,761) | 1,843^b^  (1,206-4,719) | 4,089^a^  (2,598-6,759) | 1,972^b^  (1,213-6,025) | <0.01 |
| 4-hydroxycinnamic acid | 1,636^b^  (1,255-2,227) | 8,035^a^  (4,603-23,377) | 1,419^b^  (1,027-2,517) | 1,465^b^  (1,165-2,443) | 13,352^a^  (5,072-33,944) | 1,467^b^  (625-2,431) | <0.01 |
| 4-hydroxyphenylacetic acid | 42,535^a^  (27,466-79,397) | 19,423^b^  (2,564-41,632) | 61,252^a^  (21,157-105,898) | 41,639^a^  (19,711-123,186) | 16,726^b^  (160-24,795) | 34,960^a^  (18,054-104,601) | <0.01 |
| 5-aminovaleric acid | 1,013,536^a^  (513,660-1,447,712) | 470,552^b^  (173,881-937,037) | 1,053,479^a^  (669,788-1,673,497) | 899,848^a^  (434,194-1,944,984) | 539,233^b^  (330,767-789,918) | 1,043,659^a^  (466,512-1,838,071) | <0.01 |
| 5'-deoxy-5'-methylthioadenosine | 234^b^  (167-584) | 488^a^  (82-725) | 488^b^  (106-1,201) | 186^b^  (97-719) | 434^a^  (318-718) | 180^b^  (87-466) | <0.01 |
| 6-deoxyglucose | 41,343^a^  (3,128-57,100) | 22,610^b^  (5,176-53,494) | 24,305^a^  (11,648-65,329) | 35,812^a^  (15,310-143,317) | 17,387^b^  (8,349-45,526) | 41,037^b^  (16,090-206,309) | 0.02 |
| 7-methylguanine NIST | 2,722^a^  (1,967-3,212) | 780^c^  (458-1,625) | 1,646^b^  (1,274-2,576) | 2,663^a^  (1,877-3,834) | 772^c^  (427-1,275) | 1,864^b^  (1,224-5,953) | <0.01 |
| acetophenone NIST | 9,386^a^  (6,497-14,000) | 4,886^c^  (3,738-7,634) | 7,876^b^  (4,226-12,411) | 7,671^a^  (4,030-13,393) | 5,340^c^  (3,032-7,283) | 7,541^b^  (1,901-12,860) | <0.01 |
| adenosine | 848^a^  (584-1,624) | 316^c^  (142-691) | 528^b^  (321-1,095) | 732^a^  (306-1,520) | 305^c^  (241-538) | 755^b^  (222-2,658) | <0.01 |
| alpha-aminoadipic acid | 1,503^a^  (1,093-2,083) | 695^b^  (391-1,247) | 713^b^  (530-996) | 1,331^a^  (407-1,930) | 689^b^  (339-881) | 792^b^  (319-1,266) | <0.01 |
| arachidonic acid | 23,156^a^  (14,147-92,848) | 8,335^c^  (3,867-36,152) | 13,338^b^  (8,093-26,999) | 14,008^a^  (8,246-31,402) | 7,011^c^  (2,391-20,560) | 14,078^b^  (9,267-22,979) | <0.01 |
| asparagine | 2,142^b^  (1,927-3,293) | 7,681^a^  (2,166-14,133) | 3,243^b^  (1,147-3,767) | 2,486^b^  (1,693-5,906) | 6,089^a^  (4,651-13,658) | 1,757^b^  (1,358-5,776) | <0.01 |
| benzoic acid | 18,248^a^  (11,999-36,487) | 7,654^b^  (4,502-12,055) | 24,009^a^  (9,406-43,503) | 15,174^a^  (11,205-48,217) | 6,507^b^  (5,124-11,153) | 17,509^a^  (14,244-55,367) | <0.01 |
| beta-alanine | 62,120^a^  (30,295-179,463) | 1,201^c^  (243-26,018) | 53,238^b^  (4,494-87,800) | 69,881^a^  (23,202-203,168) | 1,216^c^  (636-28,256) | 27,005^b^  (8,188-47,976) | <0.01 |
| beta-sitosterol | 50,213^a^  (38,234-78,002) | 29,540^b^  (16,023-41,027) | 46,337^a^  (24,089-75,619) | 47,660^a^  (33,580-136,596) | 34,755^b^  (22,485-54,253) | 46,616^a^  (22,223-74,152) | <0.01 |
| biphenyl | 3,169^a^  (1,833-6,573) | 820^c^  (470-1,651) | 1,712^b^  (791-2,449) | 2,399^a^  (1,334-3,831) | 1,056^c^  (629-1,806) | 1,111^b^  (775-4,216) | <0.01 |
| butyrolactam NIST | 6,568^a^  (4,141-17,913) | 2,789^c^  (1,935-5,064) | 4,346^b^  (2,453-5,128) | 5,853^a^  (4,049-11,196) | 3,469^c^  (1,678-5,100) | 3,629^b^  (2,463-8,860) | <0.01 |
| capric acid | 2,424^a^  (1,302-4,204) | 755^b^  (421-1,477) | 2,260^a^  (1,375-4,132) | 1,997^a^  (1,090-3,411) | 643^b^  (482-932) | 1,438^a^  (764-3,002) | <0.01 |
| caprylic acid | 7,925^a^  (4,085-18,897) | 2,079^b^  (996-5,962) | 9,830^a^  (5,314-21,635) | 5,725^a^  (2,097-19,799) | 1,643^b^  (947-2,364) | 7,987c  (1,743-13,781) | <0.01 |
| catechol | 2,078^a^  (890-3,538) | 240^b^  (137-1,975) | 1,472^a^  (255-2,892) | 1,908^a^  (685-2,994) | 270^b^  (157-458) | 2,283^a^  (961-3,188) | <0.01 |
| cellobiose | 15,190^a^  (5,457-29,788) | 5,109^b^  (1,738-43,182) | 5,063^b^  (2,557-12,603) | 13,562^a^  (2,201-25,493) | 4,212^b^  (2,808-89,792) | 10,658^b^  (1,087-20,781) | 0.01 |
| cholic acid | 2,933^c^  (1,456-13,399) | 62,504^a^  (1,494-318,640) | 9,197^b^  (1,883-71,273) | 2,849^c^  (1,221-13,634) | 110,785^a^  (43,650-229,826) | 6,631^b^  (1,267-95,346) | <0.01 |
| cis-gondoic acid | 742^a^  (368-1,312) | 369^b^  (155-740) | 709^a^  (246-1,501) | 454^a^  (332-1,685) | 272^b^  (168-359) | 598^a^  (314-848) | <0.01 |
| conduritol-beta-epoxide | 372^c^  (301-659) | 127,855^a^  (32,474-299,529) | 644^b^  (274-1,096) | 599^c^  (339-2,079) | 105,561^a^  (63,902-274,817) | 665^b^  (410-3,903) | <0.01 |
| creatinine | 1,696^b^  (736-14,181) | 9,255^a^  (6,446-52,304) | 2,098^b^  (1,014-14,103) | 3,258^b^  (762-24,666) | 9,668^a^  (4,177-23,425) | 5,237^b^  (1,485-10,277) | <0.01 |
| cysteine | 2,796^b^  (2,143-4,139) | 10,173^a^  (3,849-16,922) | 2,223^c^  (1,086-3,496) | 2,538^b^  (738-3,327) | 14,529^a^  (5,405-26,048) | 1,597^c^  (1,095-3,150) | <0.01 |
| cystine | 1,085^a^  (582-1,608) | 968^a^  (552-7,458) | 474^b^  (249-3,602) | 1,184^a^  (596-2,080) | 1,743^a^  (463-3,600) | 969^b^  (547-1,528) | 0.03 |
| diglycerol | 4,894^a^(3,212-6,336) | 5,556^a^  (889-7,519) | 3,628^b^  (808-6,204) | 4,578^a^  (434-13,306) | 6,465^a^  (3,406-8,845) | 3,477^b^  (2,385-7,822) | <0.01 |
| dihydrocholesterol | 1,960^a^  (1,360-2,384) | 870^c^  (630-1,538) | 1,634^b^  (923-2,355) | 1,659^a^  (1,017-4,691) | 774^c^  (556-1,369) | 1,225^b^  (253-2,462) | <0.01 |
| erythrose | 413^a^  (337-663) | 464^a^  (283-799) | 309^b^  (142-518) | 449^a^  (227-3,179) | 568^a^  (151-979) | 287^b^  (155-2,775) | <0.01 |
| ethanolamine | 42,429^b^  (264-74,837) | 71,841^a^  (570-165,616) | 564^c^  (261-46,814) | 58,925^b^  (401-95,497) | 104,054^a^  (351-229,082) | 32,882^c^  (324-57,779) | <0.01 |
| fructose | 9,225^a^  (1,964-36,517) | 2,600^b^  (332-21,509) | 2,315^b^  (425-6,072) | 12,533^a^  (3,479-22,024) | 1,004^b^  (324-25,857) | 2,556^b^  (79-8,828) | <0.01 |
| glucose | 197,796^a^  (48,781-355,279) | 48,135^c^  (8,246-280,766) | 39,124^b^  (18,291-459,260) | 125,159^a^  (82,657-286,126) | 29,508^c^  (9,810-149,126) | 134,083^b^  (14,245-646,042) | <0.01 |
| glucose-1-phosphate | 1,593^a^  (703-2,363) | 1,518^a^  (763-7,749) | 1,407^b^  (953-1,754) | 1,970^a^  (1,184-3,500) | 1,960^a^  (1,228-3,546) | 1,251^b^  (626-1,754) | <0.01 |
| glutamine | 8,346^b^  (5,322-11,468) | 13,828^a^  (8,563-35,076) | 19,148^a^  (9,883-27,322) | 7,356^b^  (2,188-14,690) | 16,531^a^  (8,078-42,658) | 12,089^a^  (4,728-27,845) | <0.01 |
| glyceric acid | 10,168^a^  (6,868-16,060) | 7,325^b^  (3,718-13,154) | 13,287^a^  (7,625-24,975) | 11,171^a^  (6,130-40,842) | 7,034^b^  (4,246-12,467) | 9,756^a^  (5,009-23,472) | <0.01 |
| glycerol | 326,819^a^  (247,223-448,217) | 164,183^c^  (115,037-209,139) | 244,616^b^  (145,254-460,503) | 340,022^a^  (205,041-483,169) | 159,067^c^  (99,938-203,453) | 279,814^b^  (184,887-377,807) | <0.01 |
| glycine | 51,805^ab^  (40,546-71,848) | 52,452^b^  (28,982-133,434) | 73,734^a^  (47,508-91,533) | 57,562^ab^  (40,759-140,133) | 50,281^b^  (24,987-295,600) | 65,474^a^  (51,475-127,185) | 0.02 |
| glycolic acid | 20,613^a^  (14,181-32,040) | 8,204^c^  (3,722-18,919) | 14,165^b^  (6,864-20,780) | 18,477^a^  (444-41,801) | 8,430^c^  (3,199-17,034) | 13,192^b^  (702-20,144) | <0.01 |
| guanosine | 492^a^  (131-1,681) | 154^b^  (83-607) | 264^a^  (201-867) | 671^a^  (215-5,048) | 127^b^  (80-228) | 855^a^  (206-3,843) | <0.01 |
| heptadecanoic acid | 24,665^a^  (17,726-33,067) | 14,110^b^  (8,750-25,240) | 20,563^a^  (14,720-33,636) | 19,958^a^  (14,365-30,429) | 13,697^b^  (5,712-27,071) | 18,772^a^  (13,356-28,753) | <0.01 |
| hexitol | 899^b^  (427-1,537) | 1,517^a^  (287-4,726) | 646^b^  (240-1,403) | 721^b^  (383-1,910) | 1,590^a^  (363-3,022) | 693^b^  (390-1,986) | 0.02 |
| hexuronic acid | 2,272^b^  (648-4,666) | 2,548^a^  (726-78,735) | 2,566^b^  (1,204-6,708) | 1,616^b^  (339-5,720) | 5,424^a^  (1,166-31,188) | 2,237^b^  (998-5,354) | 0.02 |
| homocystine | 1,045^b^  (347-3,209) | 625^b^  (269-1,098) | 2,315^a^  (461-3,728) | 674^b^  (218-2,579) | 762^b^  (549-1,535) | 1,071^a^  (259-3,091) | 0.01 |
| homoserine | 4,119^a^  (3,590-6,226) | 2,458^b^  (1,830-4,301) | 4,052^a^  (3,061-6,056) | 4,262^a^  (2,951-7,620) | 2,232^b^  (1,845-3,116) | 3,916^a^  (1,986-7,098) | <0.01 |
| hydroxycarbamate NIST | 14,344^a^  (5,838-29,949) | 6,175^c^  (3,619-14,668) | 11,544^b^  (4,863-17,413) | 13,236^a^  (3,363-18,374) | 5,076^c^  (3,510-11,583) | 6,971^b^  (2,437-17,393) | <0.01 |
| hypoxanthine | 41,758^a^  (13,085-72,828) | 9,432^c^  (2,749-27,767) | 28,212^b^  (4,848-51,817) | 41,821^a^  (17,140-69,697) | 9,785^c^  (2,386-26,489) | 32,496^b^  (1,575-56,026) | <0.01 |
| indole-3-acetate | 20,743^a^  (14,077-23,638) | 3,828^b^  (1,115-9,503) | 22,084^a^  (12,120-48,972) | 18,542^a^  (10,247-27,294) | 2,643^b^  (1,675-4,983) | 18,757^a^  (11,691-99,336) | <0.01 |
| indole-3-lactate | 115,052^a^  (74,011-172,000) | 34,761^c^  (16,259-45,655) | 48,501^b^  (19,622-126,079) | 93,617^a^  (46,576-150,970) | 34,770^c^  (17,743-46,913) | 71,855^b^  (19,782-133,294) | <0.01 |
| inositol-4-monophosphate | 487^a^  (323-868) | 126^c^  (66-589) | 322^b^  (174-631) | 522^a^  (196-1,203) | 102^c^  (83-291) | 350^b^  (178-667) | <0.01 |
| isoheptadecanoic acid N | 7,860^a^  (4,863-11,157) | 2,836^c^  (433-5,810) | 5,143^b^  (4,673-9,466) | 4,856^a^  (3,409-15,214) | 2,076^c^  (1,468-3,554) | 4,963^b^  (2,550-9,784) | <0.01 |
| isopentadecanoic acid | 53,407^a^  (28,784-91,274) | 24,171^c^  (15,867-34,465) | 29,321^b^  (4,722-51,680) | 51,526^a^  (25,488-95,283) | 16,855^c^  (6,681-316,781) | 30,903^b^  (16,922-70,276) | <0.01 |
| isothreonic acid | 2,266^b^  (600-7,304) | 794^c^  (171-1,967) | 5,760^a^  (438-16,839) | 1,317^b^  (803-2,779) | 1,163^c^  (647-2,155) | 2,639^a^  (390-9,361) | <0.01 |
| ketohexose | 492^a^  (470-1,526) | 315^b^  (157-633) | 300^b^  (221-1,485) | 679^a^  (279-1,395) | 301^b^  (215-542) | 399^b^  (210-2,164) | <0.01 |
| kynurenic acid | 827^b^  (541-1,176) | 6,188^a^  (388-18,895) | 596^b^  (318-1,618) | 690^b^  (397-8,980) | 8,283^a^  (3,900-34,883) | 804^b^  (472-1,830) | <0.01 |
| lactamide | 199^b^  (70-276) | 538^a^  (60-2,057) | 157^b^  (86-294) | 136^b^  (80-475) | 1,158^a^  (58-2,411) | 148^b^  (71-244) | <0.01 |
| lactic acid | 580^b^  (187-136,848) | 864,555^a^  (184-1,701,564) | 36,973^b^  (136-169,063) | 524^b^  (174-79,629) | 1,322,605^a^  (117-2,202,609) | 17,542^b^  (172-125,633) | <0.01 |
| lactulose | 2,991^a^  (870-28,229) | 3,385^a^  (2,181-8,492) | 1,523^b^  (413-2,982) | 2,280^a^  (1,139-23,818) | 3,045^a^  (1,817-12,436) | 1,449^b^  (646-2,670) | <0.01 |
| lanosterol | 1,533^a^  (954-1,983) | 586^c^  (457-1,107) | 865^b^  (669-1,639) | 1,164^a^  (914-2,511) | 753^c^  (408-939) | 1,249^b^  (540-5,201) | <0.01 |
| leucine | 380,450^b^  (237,056-728,304) | 786,177^a^  (557,523-1,122,887) | 659,161^a^  (379,487-897,585) | 600,866^b^  (365,049-1,292,314) | 663,222^a^  (412,068-1,146,421) | 696,279a  (458,790-1,240,767) | <0.01 |
| levoglucosan | 646^b^  (134-909) | 824^a^  (182-2,565) | 418^b^  (304-1,561) | 587^b^  (336-3,224) | 1,134^a^  (611-1,985) | 610^b^  (172-1,699) | <0.01 |
| lithocholic acid | 34,659^a^  (2,094-119,375) | 647^c^  (401-31,262) | 10,073^b^  (576-24,309) | 25,565^a^  (4,269-66,206) | 496^c^  (344-810) | 18,850^b^  (639-39,383) | <0.01 |
| lyxitol | 3,811^a^  (2,556-7,170) | 5,076^a^  (2,289-13,903) | 2,265^b^  (1,294-3,133) | 3,995^a^  (3,417-13,060) | 2,583^a^  (1,255-20,095) | 2,887^b^  (973-6,175) | <0.01 |
| lyxose | 13,942 ^a^(5,766-23,212) | 2,091^b^  (1,517-7,909) | 12,217^a^  (4,626-54,055) | 9,078^a^  (3,105-143,435) | 1,523^b^  (612-3,117) | 15,329^a^  (5,962-125,659) | <0.01 |
| maleimide | 6,641^a^  (5,066-9,402) | 5,837^a^  (3,459-16,192) | 5,443^b^  (3,817-7,904) | 5,733^a^  (1,755-8,592) | 6,520^a^  (4,964-10,154) | 4,394^b^  (324-9,453) | 0.02 |
| malonic acid | 281^b^  (225-540) | 368^a^  (152-1,031) | 312^b^  (176-746) | 278^b^  (108-347) | 387^a^  (242-696) | 182^b^  (115-877) | 0.02 |
| methionine | 24,338^b^  (13,719-53,269) | 39,743^a^  (25,052-66,836) | 38,833^a^  (19,065-66,092) | 30,529^b^  (14,502-85,672) | 38,290^a^  (24,329-56,911) | 41,506^a^  (20,462-72,222) | <0.01 |
| monomyristin | 532^a^  (327-1,882) | 218^b^  (119-1,011) | 663^a^  (259-1,568) | 639^a^  (279-1,619) | 331^b^  (92-1,344) | 545^a^  (335-1,880) | <0.01 |
| myoinositol | 15,777^b^  (5,568-69,128) | 57,317^a^  (12,635-126,493) | 11,152^c^  (4,625-46,383) | 13,998^b^  (7,156-42,586) | 54,893^a^  (21,371-89,062) | 7,815^c^  (5,020-28,775) | <0.01 |
| myristic acid | 8,918^a^  (4,368-13,416) | 3,229^b^  (1,252-7,360) | 7,937^a^  (4,469-14,142) | 6,266^a^  (3,510-10,557) | 2,063^b^  (1,257-3,442) | 7,796^a^  (1,872-10,044) | <0.01 |
| N-acetylaspartic acid | 2,783^a^  (974-4,201) | 1,437^b^  (92-4,945) | 2,625^a^  (1,072-12,085) | 2,259^a^  (507-5,585) | 1,160^b^  (366-3,315) | 2,799^a^  (820-11,850) | <0.01 |
| n-acetyl-d-hexosamine | 3,040^a^  (1,673-5,764) | 679^c^  (373-3,255) | 1,740^b^  (1,012-5,381) | 3,074^a^  (1,291-6,801) | 692^c^  (398-1,352) | 2,243^b^  (1,393-6,849) | <0.01 |
| N-acetylglycine NIST | 306^b^  (86-1,199) | 826^a^  (272-3,168) | 376^b^  (153-1,219) | 591^b^  (134-1,896) | 1,183^a^  (241-7,144) | 541^b^  (179-1,820) | 0.02 |
| N-acetylornithine | 2,667^a^  (1,805-4,052) | 957^b^  (467-2,523) | 4,150^a^  (1,827-10,553) | 2,839^a^  (1,833-89,345) | 963^b^  (630-15,246) | 2,428^a^  (1,434-6,728) | <0.01 |
| N-acetylputrescine | 20,935^a^  (4,290-47,531) | 5,452^b^  (3,188-25,530) | 24,448^a^  (5,482-57,495) | 12,568^a^  (1,630-30,108) | 8,947^b^  (3,494-18,639) | 9,465^a^  (1,983-35,398) | <0.01 |
| nicotinamide | 954^a^  (741-1,947) | 640^c^  (374-1,175) | 843^b^  (469-1,272) | 911^a^  (501-2,700) | 692^c^  (191-1,243) | 707^b^  (485-1,294) | <0.01 |
| nicotinic acid | 51,889^a^  (21,046-78,048) | 16,883^b^  (1,723-53,115) | 52,746^a^  (29,845-64,863) | 50,934^a^  (27,342-130,643) | 17,408^b^  (10,458-30,029) | 40,637^a^  (17,482-62,505) | <0.01 |
| nonadecanoic acid | 2,203^a^  (679-3,097) | 1,402^b^  (751-2,231) | 1,932^a^  (1,263-3,522) | 1,699^a^  (1,089-2,918) | 1,451^b^  (1,100-2,254) | 1,962^a^  (1,301-2,658) | 0.03 |
| norvaline | 3,502^ab^  (1,826-29,972) | 3,097^b^  (1,470-20,641) | 7,612^a^  (2,389-51,988) | 3,866^ab^  (2,260-26,389) | 2,809^b^  (1,022-18,548) | 11,213^a^  (2,901-31,733) | 0.01 |
| O-acetylserine | 1,128^a^  (757-2,043) | 668^b^  (279-1,270) | 447^b^  (296-735) | 1,308^a^  (503-2,374) | 1,082^b^  (222-1,707) | 747^b^  (383-1,896) | <0.01 |
| octadecylglycerol | 24,555^a^  (16,206-52,824) | 7,069^c^  (1,765-26,719) | 13,047^b^  (4,930-24,028) | 15,078^a^  (8,121-72,070) | 3,135^c^  (584-9,592) | 10,932^b^  (5,065-26,297) | <0.01 |
| oleamide NIST | 7,699^a^  (1,864-11,067) | 3,129^b^  (1,160-7,553) | 5,420^a^  (3,666-9,303) | 5,443^a^  (615-19,663) | 2,405^b^  (621-4,236) | 6,656^a^  (3,150-10,313) | <0.01 |
| oleic acid | 40,401^b^  (24,749-114,776) | 15,738^c^  (2,702-39,881) | 44,981^a^  (22,855-86,915) | 26,91^b^  (19,612-89,367) | 6,704^c^  (2,435-18,016) | 49,928^a^  (29,018-61,558) | <0.01 |
| orotic acid | 517^a^  (216-1,193) | 409^b^  (142-1,147) | 273^b^  (131-1,113) | 512^a^  (129-2,689) | 398^b^  (181-702) | 395^b^  (167-627) | 0.03 |
| oxamic acid | 597^b^  (331-866) | 1,973^a^  (626-3,261) | 1,480^a^  (643-3,369) | 730^b^  (319-2,614) | 1,352^a^  (671-5,635) | 722^a^  (139-43,428) | <0.01 |
| palmitic acid | 186,792^a^  (109,842-232,335) | 88,098^b^  (66,611-177,432) | 153,922^a^  (113,437-225,163) | 132,811^a^  (97,095-184,494) | 81,433^b^  (48,055-150,374) | 135,200^a^  (68,296-193,914) | <0.01 |
| palmitoleic acid | 3,584^a^  (1,278-10,054) | 748^b^  (268-3,179) | 3,330^a^  (1,981-7,794) | 1,990^a^  (1,408-7,326) | 687^b^  (275-1,351) | 2,986^a^  (1,604-5,306) | <0.01 |
| pantothenic acid | 9,224^a^  (1,486-17,252) | 4,200^b^  (1,669-7,606) | 10,071^a^  (1,835-19,869) | 13,471^a^  (2,079-25,633) | 4,264^b^  (2,535-9,403) | 7,503^a^  (892-21,690) | <0.01 |
| parabanic acid NIST | 2,795^a^  (2,032-4,283) | 2,014^b^  (1,070-4,661) | 2,111^b^  (1,494-3,387) | 2,469^a^  (1,249-4,256) | 2,065^b^  (1,667-2,555) | 1,680^b^  (649-3,664) | 0.01 |
| pentadecanoic acid | 28,576^a^  (14,614-40,686) | 14,448^b^  (8,305-31,683) | 19,097^b^  (12,240-23,067) | 25,694^a^  (17,888-41,885) | 14,819^b^  (9,078-27,971) | 17,464^b^  (3,677-32,186) | <0.01 |
| pentitol | 199^b^  (152-299) | 1,654^a^  (587-4,684) | 244^b^  (105-1,398) | 212^b^  (136-326) | 2,050^a^  (481-3,961) | 205^b^  (123-2,038) | <0.01 |
| phenol | 7,378^b^  (5,229-9,743) | 19,118^ab^  (2,265-27,523) | 6,830^bc^  (3,621-12,948) | 6,139^bc^  (675-11,751) | 22,483^a^  (2,724-29,864) | 4,058^c^  (417-7,365) | <0.01 |
| phenylethylamine | 3,900^b^  (2,351-27,280) | 19,272^a^  (5,286-39,230) | 2,532^c^  (1,033-4,630) | 4,099^b^  (3,114-28,032) | 15,377^a^  (4,958-47,864) | 5,090^c^  (1,331-27,531) | <0.01 |
| pinitol | 584^b^  (210-1,155) | 547,695^a^  (61,705-842,053) | 834^b^  (417-1,255) | 833^b^  (139-5,278) | 730,027^a^  (367,028-1,298,113) | 888^b^  (515-7,129) | <0.01 |
| pipecolinic acid | 8,845^a^  (1,757-20,624) | 981^c^  (289-4,294) | 2,721^b^  (1,817-8,875) | 5,545^a^  (580-26,038) | 961^c^  (406-1,869) | 2,365^b^  (1,077-7,747) | <0.01 |
| piperidone | 36,320^a^  (17,382-109,528) | 3,220^c^  (475-68,234) | 10,357^b^  (1,690-49,145) | 23,265^a^  (228-160,453) | 4,021^c^  (598-18,384) | 20,403^b^  (5,039-75,008) | <0.01 |
| proline | 133,509^a^  (80,941-337,344) | 152,288^a^  (67,971-310,073) | 105,236^b^  (13,254-213,733) | 212,600^a^  (15,846-388,122) | 149,104^a^  (83,531-365,110) | 86,347^b^  (17,760-229,370) | 0.02 |
| propane-1,3-diol NIST | 6,582^b^  (4,250-10,253) | 9,521^a^  (2,735-17,768) | 5,215^b^  (2,976-7,953) | 4,907^b^  (1,197-11,000) | 10,327^a^  (3,009-15,544) | 4,317^b^  (531-7,219) | 0.01 |
| pseudouridine | 7,411^a^  (665-14,519) | 2,686^b^  (1,024-9,042) | 4,695^a^  (3,822-6,460) | 6,495^a^  (2,052-21,809) | 3,628^b^  (1,640-8,732) | 5,871^a^  (3,134-8,548) | <0.01 |
| quinic acid | 2,052^a^  (673-15,221) | 1,571^a^  (277-17,177) | 768^b^  (278-4,105) | 3,383^a^  (459-10,651) | 1,476^a^  (601-16,296) | 702^b^  (313-2,790) | <0.01 |
| ribitol | 3,828^a^  (1,414-7,117) | 3,371^a^  (1,323-10,704) | 1,895^b^  (995-7,490) | 3,292^a^  (2,647-13,395) | 6,539^a^  (2,152-20,497) | 3,751^b^  (1,355-4,837) | 0.04 |
| ribose | 62,250^a^  (26,873-123,237) | 13,404^b^  (4,062-39,258) | 74,287^a^  (23,481-143,096) | 54,956^a^  (3,469-90,336) | 8,703^b^  (1,455-19,589) | 68,870^a^  (11,458-143,402) | <0.01 |
| saccharic acid | 157^b^  (94-207) | 420^a^  (29-978) | 170^b^  (94-362) | 125^b^  (96-221) | 556^a^  (172-1,430) | 190^b^  (79-268) | <0.01 |
| salicylic acid | 803^a^  (444-1,066) | 367^b^  (197-567) | 657^a^  (374-1,149) | 745^a^  (562-1,351) | 336^b^  (230-524) | 715^a^  (491-966) | <0.01 |
| serine | 63,703^b^  (29,819-122,892) | 135,875^a^  (75,099-274,958) | 72,686^b^  (21,262-114,377) | 102,893^b^  (25,197-169,286) | 102,119^a^  (64,885-217,478) | 73,770^b^  (41,141-139,484) | <0.01 |
| stearic acid | 908,765^a^  (597,496-1,188,664) | 669,665^b^  (337,690-944,122) | 827,367^a^  (517,681-1,234,980) | 682,794^a^  (407,260-1,132,504) | 568,348^b^  (408,060-936,059) | 725,122^a^  (536,713-1,148,097) | 0.01 |
| sucrose | 18,326^a^  (3,837-149,019) | 4,540^b^  (392-82,218) | 6,326^b^  (1,263-45,597) | 30,478^a^  (3,913-95,227) | 6,982^b^  (1,113-87,891) | 6,431^b^  (1,766-26,368) | 0.01 |
| taurine | 804^c^  (378-5,520) | 10,440^a^  (3,648-23,458) | 5,364^b^  (497-15,589) | 796^c^  (294-28,965) | 6,536^a^  (916-23,038) | 1,914^b^  (88-18,532) | <0.01 |
| threitol | 330^b^  (136-1,359) | 1,565^a^  (150-2,298) | 448^b^  (124-2,446) | 260^b^  (132-4,115) | 2,103^a^  (134-2,709) | 568^b^  (208-1,581) | <0.01 |
| threonic acid | 1,396^ab^  (369-3,194) | 707^b^  (355-1,367) | 2,400^a^  (738-3,391) | 786^ab^  (184-1,927) | 971^b^  (607-1,862) | 1,360^a^  (256-4,355) | 0.04 |
| threonine | 25,605^b^  (19,341-51,332) | 60,426^a^  (39,018-110,451) | 37,209^b^  (18,546-53,962) | 29,621^b^  (20,353-52,527) | 73,101^a^  (29,743-108,810) | 28,413^b^  (21,494-45,582) | <0.01 |
| tocopherol alpha- | 95,816^a^  (73,760-138,975) | 32,206^c^  (15,570-52,885) | 73,129^b^  (30,198-133,703) | 62,421^a^  (51,776-260,882) | 28,997^c^  (17,471-43,058) | 61,680^b^  (29,189-100,815) | <0.01 |
| tocopherol delta- NIST | 1,673^a^  (620-2,227) | 653^b^  (223-1,043) | 1,079^a^  (390-2,191) | 1,449^a^  (558-2,984) | 622^b^  (146-844) | 1,361^a^  (707-2,003) | <0.01 |
| tocopherol gamma- | 4,583^a^  (378-6,599) | 2,439^c^  (1,538-4,039) | 3,433^b^  (1,764-5,439) | 3,668^a^  (2,361-11,096) | 1,918^c^  (1,003-3,625) | 2,994^b^  (1,451-4,875) | <0.01 |
| tryptophan | 30,456^b^  (15,983-142,360) | 99,160^a^  (21,844-170,273) | 68,305^a^  (32,808-138,909) | 40,964^b^  (16,338-83,084) | 87,474^a^  (51,120-133,855) | 95,811^a^  (31,515-284,375) | <0.01 |
| tyrosine | 104,866^b^  (57,993-148,579) | 76,937^c^  (38,036-158,840) | 205,097^a^  (99,195-364,962) | 132,772^b^  (53,707-226,568) | 79,714^c^  (51,338-181,230) | 132,797^a^  (53,054-285,476) | <0.01 |
| tyrosol | 1,916^b^  (1,576-7,211) | 3,609^a^  (2,265-10,507) | 1,130^c^  (705-5,362) | 2,583^b^  (1,571-5,053) | 5,008^a^  (1,200-14,591) | 1,629^c^  (997-3,129) | <0.01 |
| uracil | 152,319^a^  (98,477-242,866) | 23,059^c^  (14,976-85,914) | 97,800^b^  (58,191-155,664) | 169,808^a^  (128,816-314,153) | 22,677^c^  (13,337-43,226) | 128,071^b^  (79,713-260,607) | <0.01 |
| uric acid | 7,446^a^  (1,093-8,587) | 3,464^b^  (154-12,288) | 6,690^a^  (1,699-14,503) | 5,013^a^  (1,958-16,664) | 821^b^  (150-6,099) | 3,624^a^  (1,048-12,261) | <0.01 |
| valine | 276,891^b^  (188,238-400,987) | 521,913^a^  (339,446-937,706) | 488,616^a^  (189,841-633,644) | 344,086^b^  (87,249-645,178) | 452,652^a^  (260,008-849,062) | 370,097^a^  (152,157-754,861) | <0.01 |
| vanillic acid | 733^b^  (326-1,558) | 1,979^a^  (451-3,611) | 1,359^a^  (605-7,199) | 859^b^  (504-11,076) | 2,460^a^  (1,577-3,727) | 1,725^a^  (458-7,575) | <0.01 |
| xanthine | 43,762^a^  (34,411-66,584) | 10,772^c^  (4,689-33,086) | 34,329^b^  (16,101-43,776) | 44,327^a^  (27,891-69,856) | 9,625^c^  (4,558-53,558) | 34,764^b^  (9,927-64,018) | <0.01 |
| xylitol | 933^b^  (331-1,426) | 1,526^a^  (269-7,764) | 612^b^  (366-1,548) | 774^b^  (519-1,567) | 2,836^a^  (732-8,855) | 850^b^  (489-1,431) | <0.01 |
| xylose | 231,751^a^  (104,411-364,529) | 24,380^b^  (7,405-151,201) | 210,772^a^  (81,780-580,529) | 162,564^a^  (66,841-451,006) | 15,227^b^  (1,119-134,572) | 214,985^a^  (108,384-651,555) | <0.01 |
| xylulose NIST | 6,002^a^  (1,846-12,519) | 641^b^  (370-4,034) | 4,859^a^  (2,175-26,124) | 3,135^a^  (1,569-9,975) | 718^b^  (287-3,330) | 4,970^a^  (322-26,414) | <0.01 |
